# A [FeFe] Hydrogenase–Rubrerythrin Chimeric Enzyme Functions to Couple H_2_ Oxidation to Reduction of H_2_O_2_ in the Foodborne Pathogen *Clostridium perfringens*

**DOI:** 10.1021/jacs.4c18425

**Published:** 2025-03-06

**Authors:** Jesse Taylor, David W. Mulder, Patrick S. Corrigan, Michael W. Ratzloff, Natalia Irizarry Gonzalez, Carolyn E. Lubner, Paul W. King, Alexey Silakov

**Affiliations:** † Department of Chemistry, Pennsylvania State University, University Park, Pennsylvania 16802, United States; ‡ Biosciences Center, National Renewable Energy Lab, Golden, Colorado 80401, United States

## Abstract

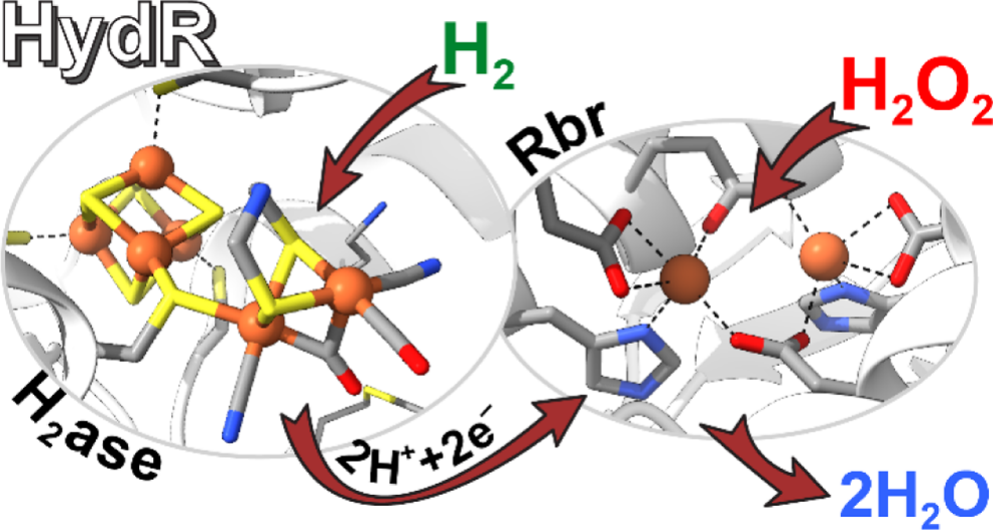

[FeFe] hydrogenases are a diverse class of H_2_-activating enzymes with a wide range of utilities in nature. As H_2_ is a promising renewable energy carrier, exploration of the increasingly realized functional diversity of [FeFe] hydrogenases is instrumental for understanding how these remarkable enzymes can benefit society and inspire new technologies. In this work, we uncover the properties of a highly unusual natural chimera composed of a [FeFe] hydrogenase and rubrerythrin as a single polypeptide. The unique combination of [FeFe] hydrogenase with rubrerythrin, an enzyme that functions in H_2_O_2_ detoxification, raises the question of whether catalytic reactions, such as H_2_ oxidation and H_2_O_2_ reduction, are functionally linked. Herein, we express and purify a representative chimera from *Clostridium perfringens* (termed *Cper*HydR) and apply various electrochemical and spectroscopic approaches to determine its activity and confirm the presence of each of the proposed metallocofactors. The cumulative data demonstrate that the enzyme contains a surprising array of metallocofactors: the catalytic site of [FeFe] hydrogenase termed the H-cluster, two [4Fe-4S] clusters, two rubredoxin Fe(Cys)_4_ centers, and a hemerythrin-like diiron site. The absence of an H_2_-evolution current in protein film voltammetry highlights an exceptional bias of this enzyme toward H_2_ oxidation to the greatest extent that has been observed for a [FeFe] hydrogenase. Here, we demonstrate that *Cper*HydR uses H_2_, catalytically split by the hydrogenase domain, to reduce H_2_O_2_ by the diiron site. Structural modeling suggests a homodimeric nature of the protein. Overall, this study demonstrates that *Cper*HydR is an H_2_-dependent H_2_O_2_ reductase. Equipped with this information, we discuss the possible role of this enzyme as a part of the oxygen-stress response system, proposing that *Cper*HydR constitutes a new pathway for H_2_O_2_ mitigation.

## Introduction

Hydrogenases are enzymes that catalyze the uptake or evolution of H_2_.^1^ These enzymes play a critical role in H_2_ metabolism in organisms and are widespread in nature.^2−4^ In this work, we focus on [FeFe] hydrogenases, a superfamily of structurally diverse enzymes that typically demonstrate bidirectional activity, i.e., capable of reducing H^+^ and oxidizing H_2_.^5^ The common gene designation is *hydA*, although several well-studied enzymes of this family bear names that do not follow the gene-based naming conventions for historical reasons.^6^

The versatility of [FeFe] hydrogenases in nature appears to enable a wide range of functions, although only a small subset of examples have been explored so far.^1,5^ Some of these enzymes have exceptional efficiency in producing H_2_ with turnovers >10,000 s^–1^,^7−9^ while others demonstrate a bias toward H_2_ oxidation.^7,10^ Many [FeFe] hydrogenases studied to date act as electron sinks to control the buildup of reducing equivalents, e.g., reduced ferredoxins, originating from energetic metabolic processes. Such enzymes typically exhibit high H_2_ production rates, making them promising targets for bio-H_2_-production applications.^11,12^

H_2_-uptake [FeFe] hydrogenases are much less well investigated. The most explored representative is the [FeFe] hydrogenase 2 from *Clostridium pasteurianum* (commonly known as *Cp*II).^7,13−15^ It is an H_2_-uptake enzyme that functions to oxidize H_2_, which is produced as a byproduct during N_2_-fixation by the nitrogenase enzyme.^10,15,16^ It is worth noting that *Cp*II exhibits both H_2_-uptake (positive) and H_2_-evolution (negative) current in protein film voltammetry (PFV) experiments.^7^ However, the proportions of positive and negative currents differ from the more H_2_-evolution-active *Cp*HydA1 (or *Cp*I). *Cp*II requires a negative overpotential to display substantial H_2_-evolution in PFV.

In some cases, [FeFe] hydrogenases exist as a part of multimeric enzymatic complexes. Most notably, in 2013, Schuchmann and Müller reported the first characterization of an H_2_-dependent carbon dioxide reductase (HDCR) in *Acetobacterium woodii*.^17^ This protein complex comprises four subunits: an [FeFe] hydrogenase, formate dehydrogenase, and two electron-transfer subunits. Later, a similar complex was identified in a thermophile *Thermoanaerobacter kivui*.^18−20^ Both HDCRs demonstrated exceptional turnover frequency for hydrogenation of CO_2_, as well as formate-dependent H_2_ evolution.

The emergence of HDCR and other functional subclasses of [FeFe] hydrogenases, such as sensing^21,22^ and bifurcating^23−26^ enzymes, uncover the diversity of biochemical functions for [FeFe] hydrogenases in nature. Notably, HDCRs showcase the potential utilization of H_2_ as an energy source for powering chemical transformations via an [FeFe] hydrogenase as the H_2_ catalyst. Therefore, learning from such systems is paramount for creating rational design strategies for developing novel enzymatic systems that can drive important chemical transformations using H_2_ as an alternative energy source.

As inspiration for this work, we recognize earlier bioinformatic analysis of clostridial organisms by Calusinska et al.^2,3^ that predicts a wide range of functional possibilities for these enzymes along with a variety of distinct structural compositions. Among the identified constructs, the authors proposed the existence of a natural chimera containing an [FeFe] hydrogenase domain (HydA) and a rubrerythrin (Rbr) in a single polypeptide. Due to the lack of an established designation in the literature, we term this enzyme **HydR**. This case stands out for two primary reasons.

First, this enzyme represents a unique case of a direct fusion of [FeFe] hydrogenase with another catalytic domain in one polypeptide. The known multifunctional enzymatic systems are typically multimeric complexes, e.g., class I ribonucleotide reductases, carbon monoxide dehydrogenase/acetyl-CoA synthase, and nitrogenase, to name a few. To our knowledge, no reports exist of a metalloprotein encoding a HydA-domain along with another catalytic metal-containing domain in the same protein. A recent report on the potential coexistence of [NiFe]-hydrogenase and [FeFe] hydrogenase domains is, perhaps, the closest existing example.^27^

Second, the typical role of an Rbr is to catalyze the reduction of hydrogen peroxide (H_2_O_2_), coupled to low potential electrons from the pyridine nucleotide pool, as components of an oxidative stress response in strict anaerobes.^28−31^ It is, thus, intriguing that such a subunit is fused to an [FeFe] hydrogenase, since reactive oxygen species (ROS), such as H_2_O_2_, can cause irreversible degradation of its active center, H-cluster.^32,33^

**Figure 1:**
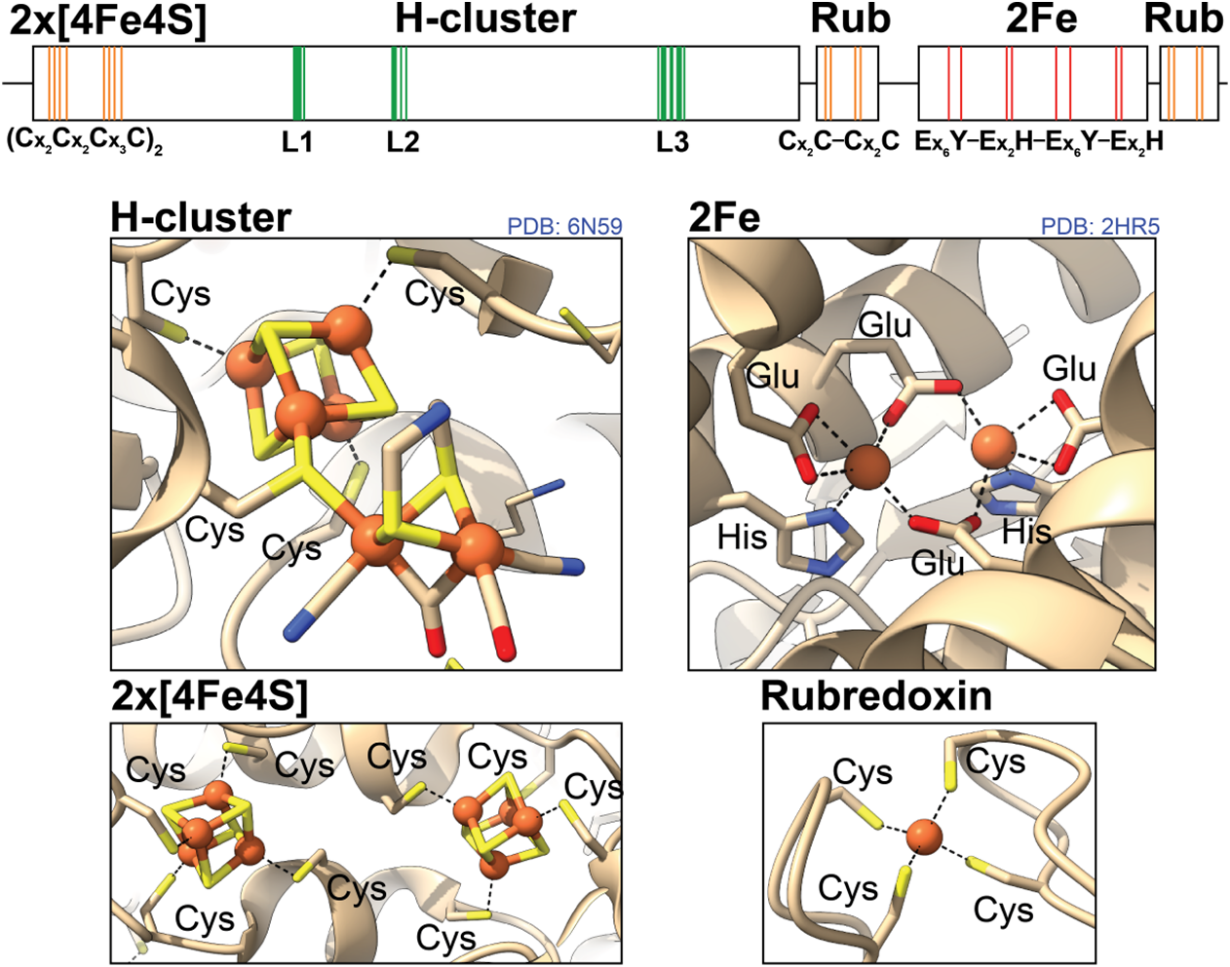
Schematic representation of *Cper*HydR domain structure (top) and the depiction of the postulated metallocofactors (bottom four panels) using available crystallograpgic data. L1 = (TSCCPxW), L2 = (M/TPCx_2_Kx_2_E) and L3 = (ExMACx_2_GCx_2_GGGP) are conserved H-cluster binding motifs.^6^ The representation of [FeFe] hydrogenase-specific cofactors (H-cluster and 2x[4Fe4S]) is based on the structure of *Cp*I (PDB 6N59) and of rubrerythrin-specific cofactors (2Fe and Rubredoxin) is based on the structure of *Pf*Rbr (PDB 2HR5).

H-cluster coordinates a biologically unique, six-iron, six-sulfur, organometallic cofactor (see Figure 1). *In vitro*, reconstitution of the H-cluster can be achieved by incubating the apo form of the enzyme with a synthetic precursor to the [2Fe]_H_ subcluster, [Fe_2_(μ-adt)(CO)_4_(CN)_2_]^2–^ (adt = azadithiolate). The semisynthetic approach simplifies the heterologous expression of these enzymes significantly and can improve protein yields.^34−37^ Additionally, many [FeFe] hydrogenases have accessory electron-transfer Fe–S clusters (termed F-clusters), most commonly of the [4Fe-4S] or [2Fe-2S] types.^1,3,7,38,39^

Rbrs, per LeGall et al.,^40^ are proteins containing a rubredoxin (Rub) and a di-iron (hemerythrin-like) center. The active di-iron center of Rbr within a four-helix bundle is coordinated by four Glu and two His residues (see Figure 1). This structure is similar among ferritin-like domain-containing enzymes, e.g., hemerythrins, nigerythrins, soluble methane monooxygenases (sMMO), and class I ribonucleotide reductases (RNR).^41,42^ One C- or N-terminal Rub domain in Rbrs stores and transfers a reducing equivalent during turnover.^41,43^ Rub contains a four-Cys coordinated monoiron, where the iron-tetrathiolate acts as an electron transporter (see Figure 1). Natively, the second reducing equivalent for the two-electron reduction of the diiron site is delivered by a standalone Rub.^29,31^ It was demonstrated that Rbrs catalytically reduce H_2_O_2_ to water on a millisecond time scale.^29^ Consequently, a natural fusion of Rbr and HydA enzymes into HydR suggests that a fast (<ms) oxidation of H_2_ by [FeFe] hydrogenase may support the generation of low-potential reducing equivalents at rates sufficient for the fast 2-electron reduction of H_2_O_2_ into 2 H_2_O by Rbr.

In this study, we focus on HydR from *Clostridium (C.) prefringens* (gene CPF-1047). *C. prefringens* is a Gram-positive, catalase-negative spore-forming anaerobe found in soil and plants.^44^ These bacteria are also one of the most common causes of foodborne diseases due to their ability to produce a number of major and, sometimes, lethal toxins.^44−47^ According to the U.S. Centers for Disease Control and Prevention (CDC), *C. perfringens* is responsible for approximately one million cases of foodborne illness a year in the United States alone. Several reports suggest the ability of *C. perfringens* to withstand exposure to oxygen is likely a contributing factor to the pathogenicity.^48−51^ Thus, H_2_O_2_-mitigating HydR may represent an additional virulence factor that enhances the viability of *C. perfringens* under otherwise noxious (micro) oxic conditions, remediating the absence of native catalase. Additionally, *C. perfringens* has been utilized for H_2_ production with biomass waste in biohydrogen plants, raising interest in this organism from the biohydrogen production standpoint.^52,53^ This fact brings up another reason to investigate key players in its H_2_ metabolism.

Overall, *C. prefringens* contains four different presumably cytoplasmic [FeFe] hydrogenases, including *Cper*HydR (gene CPF_1076). The previously studied *Cper*HydA [FeFe] hydrogenase (gene CPF_2655), a *Cp*I homologue, is likely responsible for the observed H_2_ generation.^2,52,53^ In addition to ROS protection, it has been suggested by Morra et al. that the observed co-expression of *Cper*HydR with the H_2_-producing *Cper*HydA throughout growth in *C. perfringens* may function in H_2_ cycling. Note that, as *Cper*HydR is presumably located in the cytosol, it is unlikely to be involved in energy conservation.

The work presented here aims to verify the proposed functionality of the natural chimeric protein *Cper*HydR. Herein, we explore the heterologous expression of *Cper*HydR in *Escherichia (E.) coli*. We demonstrate the ability to mature the enzyme *in vivo* using *Ca*HydEFG maturation machinery and *in vitro* using synthetic reconstitution of the FeS cofactors. We confirm the ability of this enzyme to reduce H_2_O_2_ under H_2_ atmosphere. We also perform an extensive characterization of the metallocofactors by Fourier-transformed infrared (FTIR) and electron paramagnetic resonance (EPR) spectroscopies to confirm the presence of postulated metallocofactors. Furthermore, we demonstrate the complete bias of this enzyme toward H_2_-oxidation using PFV. The confirmation of the function of this enzyme as an H_2_-dependent H_2_O_2_ reductase allows us to discuss its potential role in the oxidative stress response of such a foodborne pathogen as *C. perfringens*.

## Results

### Expression and Isolation of *Cper*HydR

We have employed several heterologous expression strategies using *E. coli* BL21 (DE3) hosts. First, we expressed holo-*Cper*HydR anaerobically in the presence of maturation factors HydEFG from *Clostridium acetobutylicum* (*Cper*HydR^mat^, see Methods section). The H_2_ uptake activity of the as-isolated *Cper*HydR^mat^ was 41 μmol H_2_/min/mg as measured by methylene blue reduction activity assay.^7^ Surprisingly, the enzyme showed very little H_2_-evolution activity ≤3 μmol H_2_/min/mg. To improve protein yields for further spectroscopic characterization, we have also expressed apo-*Cper*HydR in the absence of maturation factors with a subsequent reconstitution of the FeS clusters and the H-cluster (see Methods section). We observed the best expression levels when we fused *hydR* gene with a C-terminalStrep-tag II, along with an N-terminal 6xHis-*SUMO* tag.^54^ The apo-*Cper*HydR expression yields in the insoluble fraction of cell lysate (*Cper*HydR^pel^) and soluble fractions (*Cper*HydR^sol^) were about 0.8 mg and ≤0.5 mg per L of culture, respectively. Like *Cper*HydR^mat^, H_2_ oxidation coupled to methylene blue reduction activity of holo-*Cper*HydR^sol^ matured with a synthetic [2Fe]_H_ precursor was 26 μmol H_2_/min/mg. We were also able to resolubilize apo-*Cper*HydR^pel^ in urea, and the protein remained soluble after the removal of urea using affinity chromatography (see Materials and Methods section). Subsequently, we have also tested the ability of *Cper*HydR^pel^ to retain FeS clusters and mature with the synthetic [2Fe]_H_ precursor. Elemental analysis of the Fe/S reconstituted apo-*Cper*HydR^pel^ and apo-*Cper*HydR^sol^ (without the H-cluster) via ICP-AES shows approximately 10.4 ± 0.8 Fe ions per protein while holo-*Cper*HydR^pel^ retained 14.0 ± 0.8 Fe ions per protein (18 Fe ions are expected, see Figure 1).

### IR Spectroscopy Confirms the Presence of the H-Cluster

**Figure 2:**
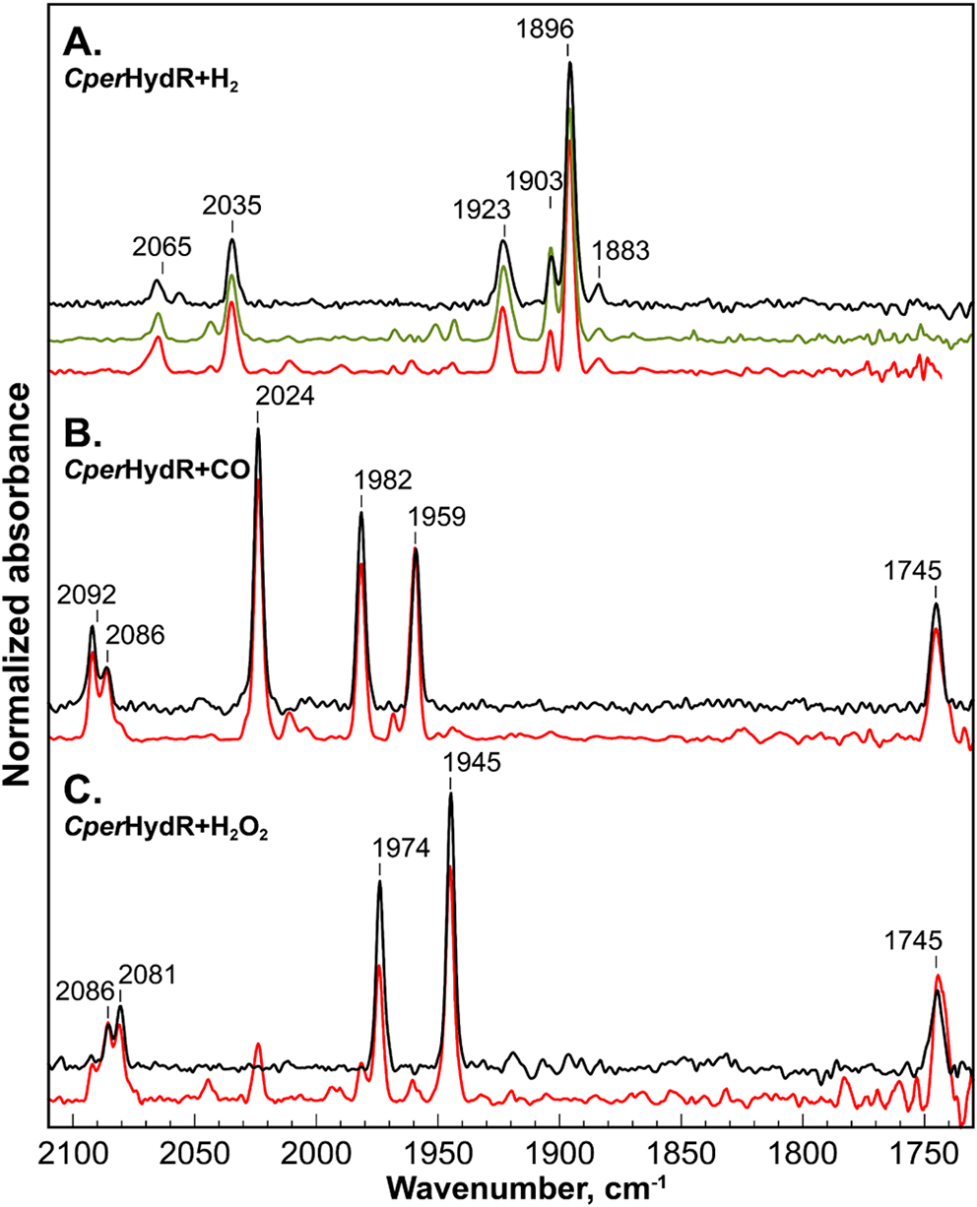
FTIR spectra of *Cper*HydR under 1 atm H_2_ (A), under 1 atm CO (B) and after incubation with H_2_O_2_ (C). Traces in red - *Cper*HydR^pel^, in black - *Cper*HydR^mat^, in green - *Cper*HydR^sol^. All spectra were acquired with 2 cm^–1^ resolution at room temperature. Spectra were normalized by the highest amplitude. Numbers above spectra indicate positions of most prominent bands.

The C–O and C–N stretch vibrations of the H-cluster are extremely sensitive to the geometric and electronic state of the H-cluster and its environment. Therefore, FTIR spectroscopy is an excellent fingerprint-like method for identifying and characterizing the oxidized and reduced states of the H-cluster (see Table S1). Remarkably, regardless of the exact method of generating the samples, we have observed near-identical spectra of *Cper*HydR^mat^, *Cper*HydR^sol,^ and *Cper*HydR^pel^ under similar conditions (see Figure 2).

The IR spectrum of the holo-*Cper*HydR^mat^ prepared under H_2_ exhibits a set of bands resembling that of reduced states of the H-cluster (see Figure 2A, black trace). Treatment of the samples with sodium dithionite resulted in similar spectra (see Figure S1A,B). Importantly, near-identical spectra were obtained for holo-*Cper*HydR^sol^ and holo-*Cper*HydR^pel^ under 1 atm H_2_ as well (see Figure 2A), indicating that the protein isolation and activation method does not affect the integrity or state of the H-cluster.

Carbon monoxide (CO) is a well-known inhibitor of [FeFe] hydrogenases that binds competitively to the open coordination site of the H-cluster. FTIR experiments demonstrate that all tested samples (*Cper*HydR^mat^ and *Cper*HydR^pel^) can be successfully incubated with CO, resulting in a well-defined 6-line spectrum (see Figure 2B). Interestingly, the low-frequency band at 1745 cm^–1^ of the μ-CO stretching vibration resembles that of *Cp*II,^7^ and is significantly downshifted compared to the μ-CO stretching vibration of prototypical group A [FeFe] hydrogenases such as *Cp*I (see Table S1).

Because of the hypothesized role of *Cper*HydR in reducing H_2_O_2_, we also incubated holo-*Cper*HydR samples with H_2_O_2_. In the presence of 218 μM H_2_O_2_, holo-*Cper*HydR^mat^ exhibits an FTIR spectrum (see Figure 2C) that closely resembles that of the H_ox_ state of *Cp*II, indicating complete oxidation of the enzyme.^7^ Similar to *Cp*II, the spectrum displayed a low-frequency CO band at 1745 cm^–1^ that can be attributed to the μ-CO stretching vibration. In the case of holo-*Cper*HydR^pel^, we have also identified a minor contribution from the H_ox_CO state, indicating some degradation of the enzymes. However, as the amount of the H_ox_CO state is comparatively small, we can conclude that the protein is relatively stable in the presence of high concentrations of H_2_O_2_. Furthermore, the similarity of the FTIR spectra of holo-*Cper*HydR^mat^ and holo-*Cper*HydR^pel^ shows that the refolded protein appears to be structurally sound, highlighting the structural stability of the protein fold of the HydA subdomain.

### Electron Paramagnetic Resonance (EPR) Measurements to Identify Metallocofactors in *Cper*HydR

**Figure 3:**
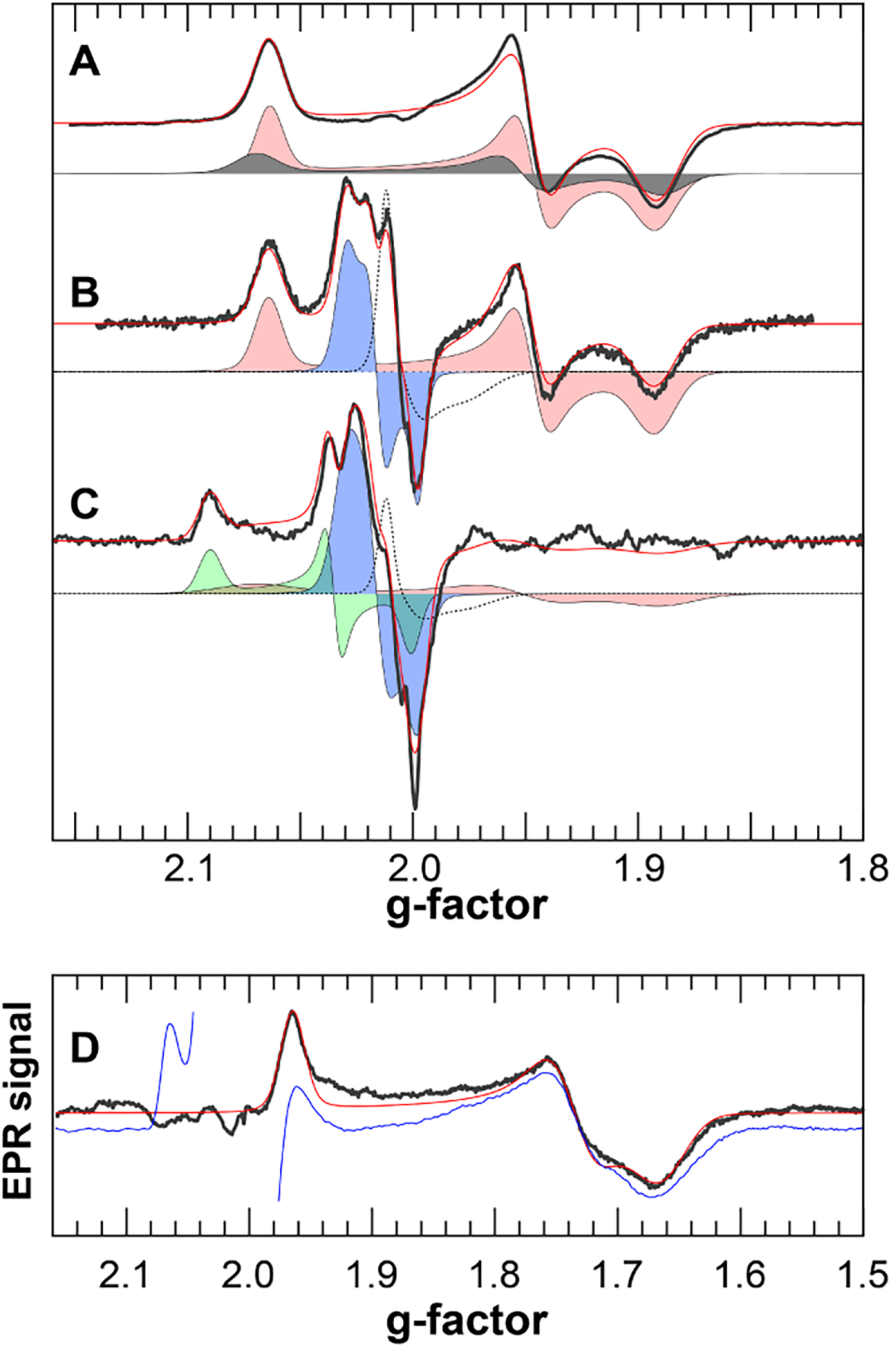
X-band CW EPR spectra of *Cper*HydR samples. **A.** As prepared *Cper*HydR^mat^ at 22K (black), **B.***Cper*HydR^pel^ under 1 atm CO measured at 10K, **C.***Cper*HydR^pel^ after incubation with H_2_O_2_ at 40K (black). In all three sections, red traces represent total simulations; shaded areas represent relevant spectral components as follows: light red and gray are [4Fe-4S]^1+^ clusters with g_1,2,3_=[2.070, 1.951, 1.889] and g_1,2,3_=[2.064, 1.946, 1.891] respectively; light blue represents the H_ox_CO state with g_1,2,3_=[2.031, 2.017, 1.996] and light green represents the H_ox_ state with g_1,2,3_=[2.090, 2.035, 2.000]. Dashed line in B and C represents the [3Fe-4S]^1+^ cluster. **D.** blue trace - *Cper*HydR^mat^ after incubation with H_2_O_2_ at 5 K, black trace is as-prepared *Cper*HydR^ΔN^, red trace–simulation of the EPR spectra of the mixed-valent diiron site with g_1,2,3_=[1.964, 1.735, 1.664]. See Table S2 for the complete set of simulation parameters. Microwave frequencies are A) 9.385 GHz, B) 9.436 GHz C) 9.435 GHz, D) 9.437 GHz (black) and 9.3781 GHz (blue).

We were also able to confirm the presence of the H_ox_ and the H_ox_CO states by EPR (see Figure 3). Just like in FTIR, the EPR signatures of the H-cluster are similar to those of *Cp*II.^7,13,14^ The H_2_O_2_-treated holo-*Cper*HydR^pel^ showed a rhombic EPR spectrum with the principal g values characteristic of the H_ox_ state. The optimum temperature for the X-band CW EPR measurements of 30–40 K is also typical for the H_ox_ state. A similar EPR spectrum was also obtained in the as-isolated sample of holo-*Cper*HydR^mat^ (see Figure S2A). Additional signals near the free-electron g value (g_e_ = 2.0023) were attributed to the mixture of EPR signals for the H_ox_CO state and [3Fe-4S]^1+^ as CO-inhibition experiments reveal similar sets of EPR signals (see Figures 2B and S3)

Sodium dithionite (NaDT)-reduced holo-*Cper*HydR^mat^ also showed a typical rhombic EPR spectrum of a [4Fe-4S]^1+^. As the H_red_ and H_red_H^+^ states dominating the NaDT-treated samples are EPR-silent,^55,56^ we assign this signal to the F-clusters. The complexity of the EPR spectrum suggests a mixture of at least two signals that somewhat differ by temperature dependence (see Figure S3). This observation is thus consistent with the postulated presence of two auxiliary [4Fe-4S] clusters.

At 5 K, the H_2_O_2_-treated holo-*Cper*HydR^mat^ sample exhibited an additional signal around g = 1.7 (see Figure 3D). The signal cannot be observed at higher temperatures, suggesting fast relaxations that cause severe spectral broadening. To verify that this signal originates from the diiron site of Rbr, we generated a HydA-domain deletion variant of *Cper*HydR, composed of only the Rbr subdomain (*Cper*HydR^ΔN^). The aerobically prepared *Cper*HydR^ΔN^ indeed shows an EPR spectrum similar to that of *Cper*HydR^mat^ (see Figure 3D). We noted that the overall signal is similar to signals observed for other Rbr homologues (see Table S3). However, some discrepancy in principal g-values likely indicates a slight structural divergence of this metallocofactor from other hemerythrin-like enzymes. We also note that at 5 K, CW EPR measurements reveal a set of low-field, high-*g*-value signals consistent with that of high-spin ferric rubredoxins (see Figure S4).

### Hydrogen Peroxide Assays Confirm the H_2_O_2_-reducing Ability of *Cper*HydR

To verify the hypothesized role of *Cper*HydR in reducing H_2_O_2_, we first tested the ability of *Cper*HydR^ΔN^ to reduce H_2_O_2_. Prereduced *Cper*HydR^ΔN^ was titrated into the solution of H_2_O_2_ in an electrochemical assay using an H_2_O_2_-sensing microelectrode.^57^

**Figure 4:**
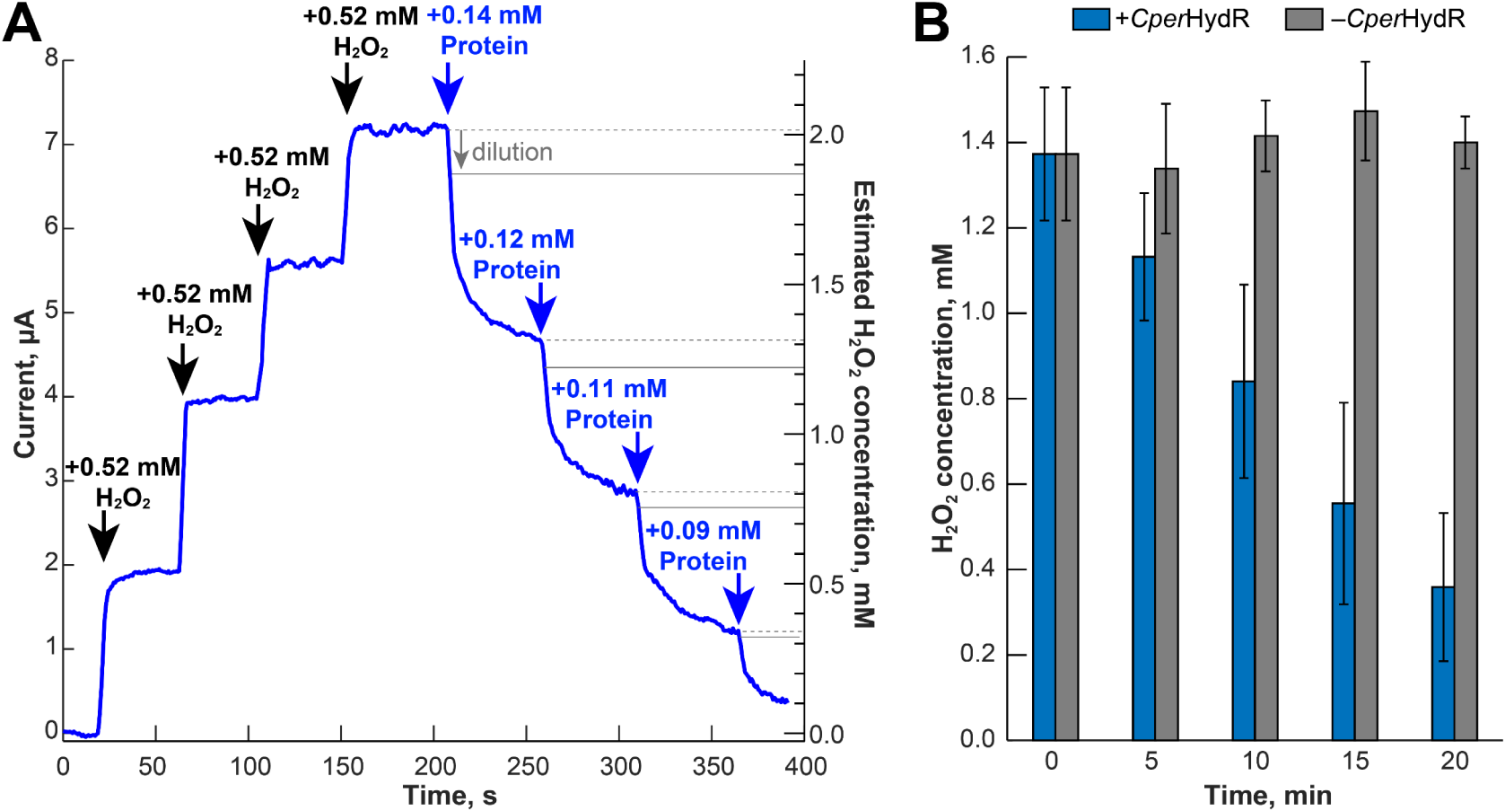
H_2_O_2_ reduction by *Cper*HydR^ΔN^ (A) and *Cper*HydR^sol^ (B). In A, numbers indicate calculated change in the concentration of H_2_O_2_ (black) and protein (blue) upon respective additions (black and blue arrows respectively). In B, blue bars indicate experiments performed in the presence of 200 nM of *Cper*HydR^sol^, gray bars designate control measuments under identical conditions but without adding protein.

Figure 4A shows an example of such an experiment, in which we stepwise titrated H_2_O_2_, followed by the titration of *Cper*HydR^ΔN^. After accounting for dilution (see calculated concentrations in Figure 4A), the initial fast phase of the reduction immediately after the addition of the protein aliquots accounts for about two molecules of H_2_O_2_ reduced per *Cper*HydR^ΔN^. The reduction matches the expected conversion stoichiometry, as fully reduced *Cper*HydR^ΔN^ can perform two turnovers by coupling the reduced rubredoxin cofactors to H_2_O_2_ reduction for the second cycle. We also observed a slower phase of the reduction that amounts to an additional 1.5–2 of H_2_O_2_ reduced per *Cper*HydR^ΔN^. These additional turnovers are likely due to a relatively slow rereduction of *Cper*HydR^ΔN^ by the probe’s counter electrode.

Encouraged by these experiments, we investigated whether the holo-*Cper*HydR^sol^ could sustain the catalytic reduction of H_2_O_2_ with H_2_. For this purpose, we incubated 200 nM of holo-*Cper*HydR^sol^ with 1.4 mM H_2_O_2_ under 1 atm H_2_, collecting samples at various time points. The residual H_2_O_2_ concentration was determined through the oxidation of reduced benzyl viologen by the collected samples. Before each measurement, we removed the enzyme from the sample using a 30 kDa concentrator to prevent side reactions with the dye. Control samples without protein underwent identical treatment. As shown in Figure 4B, the reaction resulted in a gradual decrease in H_2_O_2_ concentration, indicating catalytic turnover. After 20 min, *Cper*HydR^sol^ had reduced approximately 80% of the initial H_2_O_2_ levels. We estimate a turnover frequency of 442 ± 120 min^–1^ or a specific H_2_-uptake activity of 5.6 ± 1.5 μmol H_2_ min^–1^ mg^–1^. We have performed an additional control experiment in which we replaced hydrogen with carbon monoxide to produce an inactive yet intact holo-*Cper*HydR^pel^. No significant H_2_O_2_ reduction was observed after about 2 h under these conditions (see Figure S5).

Overall, these experiments confirm the hypothesized ability of *Cper*HydR to facilitate the reduction of H_2_O_2_ by H_2_. Consequently, we also tested for a potential O_2_-tolerance of *Cper*HydR. Even though *Cper*HydR^ΔN^ appeared to be stable in air, the wild-type *Cper*HydR exhibited oxygen sensitivity with the reduction of hydrogenase activity on par with that of [FeFe] hydrogenase I of *Clostridium acetobutylicum* (see Figure S6).

### Testing Hydrogenase Activity Using Protein Film Voltammetry

The proposed role of *Cper*HydR is to catalyze the reduction of H_2_O_2_ coupled to H_2_ oxidation. Therefore, we anticipated that this enzyme has a bias toward H_2_ oxidation. To verify this notion, we further investigated *Cper*HydR using PFV.

**Figure 5:**
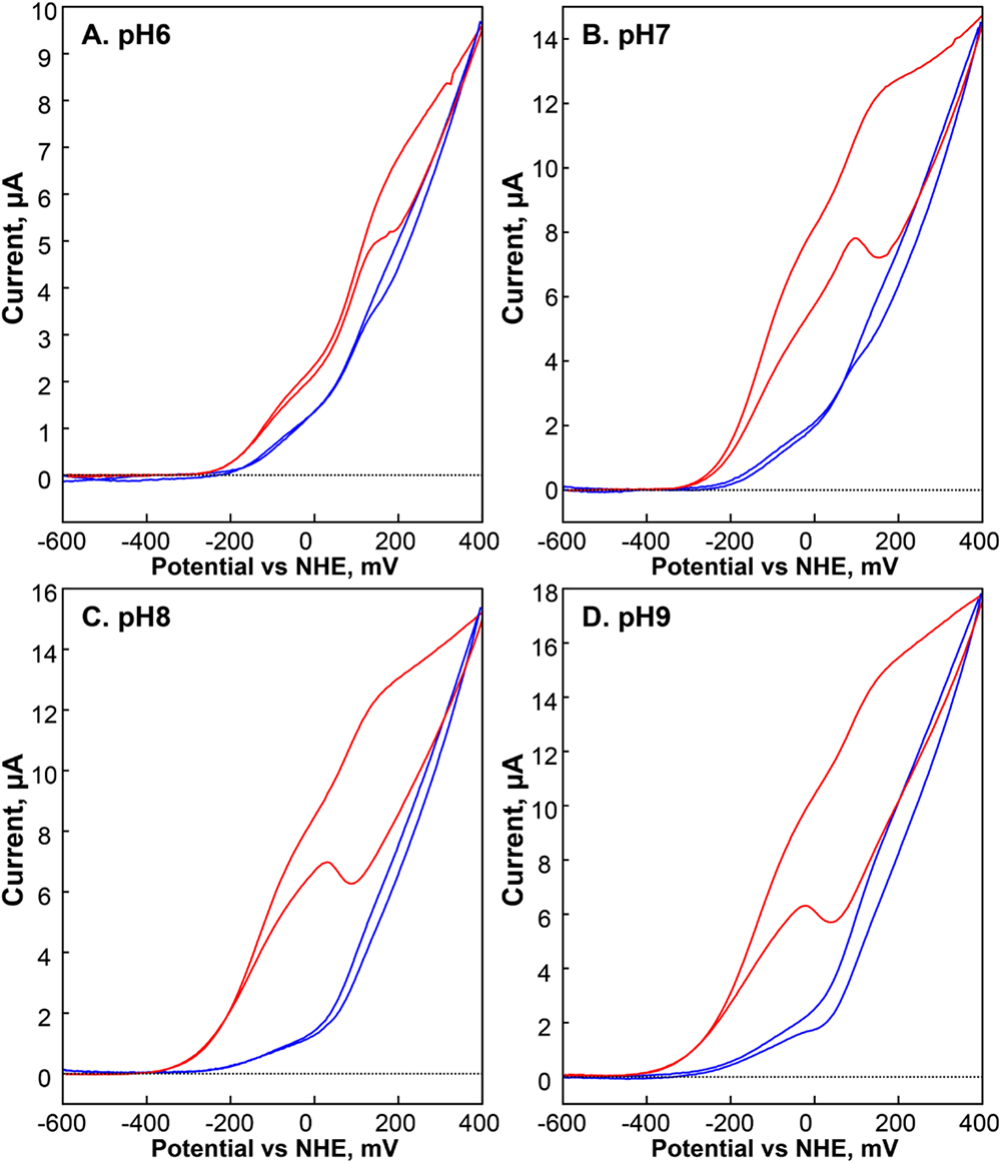
Comparison of *Cper*HydR^sol^ (blue) and *Cper*HydR^pel^(red) protein film voltammetry data at various pH. All data was acquired at 20 mV/s scan rate at room temperature. *Cper*HydR^sol^(blue traces) are scaled to match maximum current of *Cper*HydR^pel^(red traces). See original traces for all data sets in Figure S7.

For this purpose, *Cper*HydR^sol^ and *Cper*HydR^pel^ proteins were adsorbed on a rotating-disk pyrolytic graphite electrode (PGE) and tested in cyclic voltammetry under various pH conditions. Figure 5 shows the pH dependence of the holo-*Cper*HydR^sol^ and holo-*Cper*HydR^pel^ cyclic voltammetry traces (see Figure S7 for unprocessed data). Surprisingly, the experiments showed no significant (negative) H^+^-reduction current even at relatively low pH conditions, which is a stark divergence from virtually all [FeFe] hydrogenases investigated to date, including *Cp*II.^7,9,58,59^

Furthermore, there are distinct features in all the traces that appear to have different ratios in *Cper*HydR^sol^ and *Cper*HydR^pel^. In *Cper*HydR^sol^, a virtually uninhibited slope at the upper third of the voltage range dominates the traces. In the middle of the investigated range, the current traces appear to have additional contributions. In *Cper*HydR^pel^, this latter contribution is substantially more pronounced. Given the differences in sample preparations, we hypothesized that the discrepancy is structural in origin. Based on structural prediction (using AlphaFold 3^60^), proper assembly of the Rbr’s diiron site was only possible if we assumed a homodimer (see Figures S8 and S9). The monomeric Rbrs (e.g., in *Desulfovibrio vulgari*s str. Hildenborough, *Dv*Rbr^61^ differ from homodimeric Rbrs (e.g., in *Pyrococcus furiosus, Pf*Rbr^31,62^ by 13 amino acids of the flexible loop between the second and third helix. This difference appears necessary for folding the four-helix bundle within a monomer. The Rbr domain of *Cper*HydR closely matches that of the *Pf*Rbr, i.e., missing that 13-residue region (see Figure S10). Therefore, it is likely that native *Cper*HydR is a homodimer, as would be *Cper*HydR^sol^ isolated from a soluble fraction of cell lysate. Then, the procedure of solubilizing *Cper*HydR from aggregates used to prepare *Cper*HydR^pel^ may not afford an effective way to form a homodimer, producing a heterogeneous sample of monomers and dimers with the proper folding on the HydA domain but not the Rbr domain. Using size exclusion chromatography, we could demonstrate the variation in the shape of the PFV traces in different FLPC fractions (see SI) with lower molecular weight fractions containing more of *Cper*HydR^sol^-like character (see Figures S11 and S12). The absence of any catalytic current in *Cper*HydR^ΔN^ under similar conditions also rules out the possibility of the Rbr domain alone contributing significantly to the current under conditions used (see Figure S13). Therefore, this experiment provides compelling evidence that the homodimer *Cper*HydR has an exceptionally strong positive bias imposed by the presence of the di-iron site. However, even in the absence of Rbr, the HydA subdomain exhibits a near-complete bias toward H_2_ oxidation.

## Discussion

### On the Metallocofactors of *Cper*HydR

In this work, we have confirmed the postulated presence of [FeFe] hydrogenase and rubrerythin domains in *Cper*HydR using EPR and FTIR spectroscopy. Most notably, the H_ox_ and the H_ox_CO states exhibited a very low frequency of the bridging CO stretching band that has only been reported for *Cp*II so far and, thus, possibly is a hallmark of the H_2_-uptake biased [FeFe] hydrogenases.

The EPR spectrum of the di-iron site observed in H_2_O_2_-treated *Cper*HydR^mat^ and *Cper*HydR^ΔN^ appears similar to other mixed-valent Fe(II)–Fe(III) species in ferritin-like domain proteins (see Table S2). It is important to note that the S = 1/2 EPR signal in the mixed-valent Fe(II)–Fe(III) state is a result of an antiferromagnetic coupling between the high-spin ferric and ferrous sites with the apparent g-values depending on the ratio between the ferrous iron’s zero-field splitting constant and the magnitude of the exchange coupling.^40,63^ As both parameters strongly depend on the metallocofactor’s geometry, the high similarity of the diiron EPR signals in wild-type *Cper*HydR^mat^ and *Cper*HydR^ΔN^ is a good indication that the presence of the HydA domain has little to no effect on the structure of the di-iron-containing domain. Conversely, the similarity of the IR data on the H-cluster of *Cper*HydR^sol/pel^ also suggests that the HydA domain is structurally independent of the folding of the Rbr domain.

### On the Activity of *Cper*HydR

We have observed a substantial bias of *Cper*HydR toward H_2_ oxidation in dye-based activity assays and confirmed it by PFV measurements. We have also demonstrated that *Cper*HydR can catalytically reduce H_2_O_2_ using H_2_ as a reductant. Therefore, all the observations point toward the role of *Cper*HydR as an *H*_*2*_*-dependent hydroperoxide reductase*. If *Cper*HydR’s role is to act under oxidative stress, one would expect it to have some O_2_-tolerance. However, despite the expectation, *Cper*HydR is prone to O_2_-dependent degradation similar to other investigated clostridial [FeFe] hydrogenases with the exception of the O_2_-tolerant [FeFe] hydrogenases from *Clostridium beijerinkii* (*Cb*HydA1 and *Cb*A5H).^58,64,65^ It is also important to note that we did not observe any evidence of an O_2_-protected state akin to that in *Dd*HydAB and *Cb*HydA1 (H_inact_). While this is somewhat puzzling, the rate of decay appears slow. In the absence of structural data, we can only speculate that the O_2_ access to the FeS clusters (including the H-cluster) may be more hindered than in other [FeFe] hydrogenases, resulting in slower adverse oxidative reactions. The O_2_-induced degradation appears slow enough for the protein to be compatible with a microoxic environment, where the adverse effects of superoxide radicals can be effectively mitigated by superoxide dismutase (SOD). It would be interesting to see the levels of expression of SOD in comparison to *Cper*HydR in the cell. Unfortunately, no such data is available to our knowledge.

While the observed strong catalytic bias is consistent with the proposed role of *Cper*HydR, the near-complete absence of the H_2_-evolution activity is a stark departure from the typical behavior of any [FeFe] hydrogenase known to date. Even the most homologous HydA domain, *Cp*II, exhibits a noticeable H_2_-evolution current in PFV experiments at low electrochemical potentials. While the presence of the Rbr domain may hinder some proton-reduction activity, the presumably monomeric, Rbr-lacking *Cper*HydR^pel^ shows no noticeable H_2_-evolution in electrochemical experiments either.

Consequently, the observed bias may be an intrinsic property of the HydA domain of *Cper*HydR. Artz et al.^7^ proposed that two amino acids in the L2 H-cluster motif (MPCxxKxxE) control tuning the reactivity of [FeFe] hydrogenases. In the case of *Cp*I, these are Met353 near the bridging CO and Ser357 near the [4Fe-4S]_H_ subcluster (L2*^Cp^*^I^: **M**PCT**S**KKFE). The redox potential-dependent rearrangement of these two amino acids may alter the thermodynamic characteristics of the H-cluster and affect the catalytic bias. For *Cp*II, these residues are replaced with Thr and Ala (L2*^Cp^*^II^: **T**PCT**A**KKYE), possibly governing a reduction of H_2_ evolution activity. The HydA domain of *Cper*HydR is highly homologous to *Cp*II with a similar L2 motif (L2*^Cper^*^HydR^: **T**PCT**A**KKFE). This homology (see Figure S14) concurs with the similarity of the FTIR and EPR signatures of the H-cluster for *Cp*II and *Cper*HydR. It is, thus, expected that *Cper*HydR exhibits a CpII-like catalytic bias. However, the extreme nature of the bias exhibited in PFV is unprecedented, pointing to other deviations in the HydA structure between *Cp*II and *Cper*HydR, e.g., in the F-cluster region (see Figures S14 and S15). In the absence of experimentally validated structural data on either protein, we refrain from further speculations.

Also quite intriguing is the change in the catalytic overpotential depending on whether the enzyme is monomeric or dimeric. The difference in the apparent (positive) overpotential bias between oligomeric states of *Cper*HydR is an astounding 400 mV. This upshift of the catalytic current onset in PFV is not only unprecedented for [FeFe] hydrogenases but also for H_2_-uptake [NiFe] hydrogenases. It appears that the presence of the Rbr’s di-iron cofactor likely has an impact on the catalytic ability of the H-cluster, suggesting a redox cooperativity between the two sites.

### On the Physiological Role of *Cper*HydR

*C. perfringens* is annotated as a strict anaerobe but shows the ability to withstand brief exposure to air^66−68^ or even adapt to an aerobic environment for several days.^69^ This air-tolerance undoubtedly contributes to the ability of this bacterium to be a harmful foodborne pathogen.^49,51^ Unfortunately, the oxidative stress response system remains poorly understood for this bacterium. Just like most anaerobes, *C. perfringens* does not express catalase.^70^ Superoxide dismutase (SOD) and alkyl hydroperoxide reductase (AhpC) proposedly constitute the primary pathway responsible for mitigating superoxide radicals and SOD-generated H_2_O_2_, respectively.^68,71^ It was demonstrated that while *ahpC* gene expression is strongly upregulated after exposure to air, the level of *sod* gene expression is modulated much more mildly. This expression discrepancy hints at the existence of additional pathways for H_2_O_2_ mitigation. In *C. perfringens* biofilms, the upregulated expression of genes encoding glutathione peroxidase (GPx, gene CPF_0904) and hemoglobin was observed, suggesting their role in the oxidative stress response.^72,73^ Furthermore, *C. perfringens* contains several Rbr-like genes in addition to *hydR*; although, their H_2_O_2_-reducing capabilities have been questioned in the functional complementation experiments using catalase-deleted *E. coli* as a heterologous host by Jean et al.^68^

With this work, we provide evidence that *Cper*HydR is an H_2_-dependent H_2_O_2_ reductase. While we cannot rule out other possible functions, it seems likely that this enzyme acts alongside SOD because it is, presumably, the primary source of H_2_O_2_ in the cell. As for the source of H_2_, we note that the *C. perfringens* genome does not contain the nitrogen fixation system that seems to provide H_2_ for *Cp*II in *C. pasteureanum*.^7^ Instead, in *C. perfringens* H_2_ originates from the fermentative pathway containing a *Cp*I-homologue, *Cper*HydA.^70^ Morra et al. showed that the disruption of the *hydA* gene results in an abolishment of H_2_ productivity by the organism.^52^ It is also important to note that Shimizu et al. suggested that the evolution of H_2_ and CO_2_ are the primary ways *C. perfringens* establishes the anaerobic environment to help battle oxidative stress.^70^ It is also relevant to mention that an exogenous catalase can substantially improve aerobic cell viability.^74^ This observation suggests that while SOD is capable of catalyzing disproportionation of superoxide efficiently, the in-cell H_2_O_2_ mitigation pathways are inefficient under high oxidative stress conditions.

Taking all the facts together, we propose that *Cper*HydR acts within the oxidative stress response systems as a “first responder” by reducing H_2_O_2_ produced by SOD, taking advantage of the energy stored in H_2_ gas by *Cper*HydA1, while the expression of *ahpC* (and *gpx*) ramps up. A caveat to this notion is that Morra et al.^52,53^ demonstrated persistent expression of *Cper*HydR even in the early exponential growth phase where cells are not producing noticeable amounts of H_2_. However, as *Cper*HydA1 expression levels appear to persist as well, the two enzymes may operate in tandem in H_2_ cycling. Since the expression of *hydA* and *hydR* genes are downregulated in biofilms while *rbr* and *gpx* genes are upregulated,^73^ it makes us consider that the *Cper*HydR-involved H_2_O_2_ mitigation pathway is more relevant for the survival of the planktonic cells than in biofilms.

## Conclusion

In this study, we performed an extensive investigation of an unusual [FeFe] hydrogenase–rubrerythin chimeric protein from *Clostridium perfringens* heterologously expressed in *E. coli*. In our *in vitro* experiments, we were able to confirm the postulated, highly unusual composition of the metallocofactors, including the presence of the H-cluster, two [4Fe-4S] F-clusters, rubredoxin centers, and the diiron site of rubrerythin. Our investigation showed the atypically strong bias of the hydrogenase domain toward H_2_ uptake. We provide persuasive evidence that this enzyme acts as an H_2_-dependent H_2_O_2_ reductase. Overall, this work provides new insight into the utility of [FeFe] hydrogenase domains in nature and extends their functional repertoire for coupling to other catalytic reactions, such as H_2_O_2_ reduction. The findings that link H_2_ oxidation with H_2_O_2_ reduction within a fused polypeptide point to a potentially new, unrealized pathway that the dangerous foodborne pathogen can utilize to combat oxidative stress, urging further investigation of the H_2_ metabolism in this and similar organisms.

## Supporting Information Available

Materials and methods; tables comparing FTIR frequencies and EPR g values, supplementary EPR, FTIR, PFV and FPLC data; supplementary activity assays; amino acid sequence alignments; representations of structural models (PDF)
