# Supplementary material for: A [FeFe] Hydrogenase–Rubrerythrin Chimeric Enzyme Functions to Couple H2 Oxidation to Reduction of H2O2 in the Foodborne Pathogen Clostridium perfringens
Source: J Am Chem Soc. 2025 Mar 6;147(11):9764–73. doi: 10.1021/jacs.4c18425 (PMC11926857; doi:10.1021/jacs.4c18425)
Supplement: Supplementary file 1 — ja4c18425_si_001.pdf [file ja4c18425_si_001.pdf]

Supporting information for

A [FeFe] hydrogenase-rubrerythrin chimeric enzyme functions to couple H_2 oxidation to reduction of H_2O_2 in the foodborne pathogen *Clostridium perfringens*.

Jesse Taylor[1,‡], David W. Mulder[2, ‡], Patrick S. Corrigan[1], Michael W. Ratzloff[2], Natalia Irizarry Gonzalez[1], Carolyn E. Lubner[2], Paul W. King[2,*], Alexey Silakov[1*].

1 – Department of Chemistry, Pennsylvania State University, University Park, PA, 16802, USA

2 – Biosciences Center, National Renewable Energy Lab, Golden, CO, 80401, USA

‡ - These authors contributed equally: Jesse Taylor, David W. Mulder

* - corresponding authors: Paul W. King: paul.king@nrel.gov, Alexey Silakov: aus40@psu.edu

TABLE OF CONTENTS	page
**Materials and Methods**	2
**Table S1**	6
**Table S2**	7
**Table S3**	7
**Figure S1**	8
**Figure S2**	8
**Figure S3**	9
**Figure S4**	10
**Figure S5**	10
**Figure S6**	11
**Figure S7**	12
**Figure S8**	13
**Figure S9**	14
**Figure S10**	14
**Figure S11**	15
**Figure S12**	16
**Figure S13**	17
**Figure S14**	18
**Figure S15**	19
**References**	19

MATERIALS AND METHODS.

Protein expression and isolation

Protein expression and isolation of *Cper*HydR^{mat}: The entire *hydR* gene from *Clostridium perfringens* (annotated gene name CPF_1076, UniProt: A0A0H2YR94) was codon-optimized for *E. coli* and augmented with a glycine/glycine linker and C-terminal Strep-tag® II sequence (GG**WSHPQFEK**). The gene was synthesized by GeneArt and cloned into a pETDuet-1 vector (Ap^{r}) together with the *CahydE* gene.[1] The resulting vector (pETDUET-1:*Ca*hydE, *Cper*hydR) contained the C-terminal Step-tag II *CperhydR* gene at *ndeI-bglII* sites of MCSII and the *CahydE* gene at *ncoI-bamH1* sites of MCSI. Expression of in-vivo matured *Cper*HydR^{mat} was performed using procedures described elsewhere with some modifications.[1,2] Briefly, a Rosetta-2 strain of BL21(DE3) cells (Novagen) were co-transformed with pETDUET-1:*Ca*hydE, *Cper*hydR and pCDFDuet-1 (Sm^{r}) harboring *CahydF* and *CahydG* genes. Transformed cells were cultured overnight in 5 ml of TB media supplemented with 5% glycerol, 200 µg/ml carbenicillin, and 50 µg/ml streptomycin. Cultures grown overnight were diluted 1:100 into either 6x1L flasks of fresh medium or a 10L fermenter and grown at 37°C until the OD_{600} reached 0.4. The expression was induced with 1.5 mM IPTG with added 4 mM Ferric ammonium citrate, 2 mM cysteine, 0.5% glucose, and 10 mM sodium fumarate. The cultures were sparged with N_2 or Ar at 30°C overnight. The following steps were carried out under strict anaerobic conditions. The cells were harvested by centrifugation and resuspended in 90 mL buffer (50 mM Tris pH 8, 5 mM NaCl, 5% glycerol, 5 mM NaDT). The cells, supplemented 15 µL benzonase, 0.5 mL lysozyme, and 1 complete EDTA-free protease inhibitor tablet (Roche Life Sciences), were lysed by passing through French Press at 30,000 psi (Thermo Scientific) and centrifuged for 1 hour at 45,000 rpm at 4°C to separate cellular debris and cell extract. The following steps were carried out in an MBraun glove box under N_2 atmosphere. *Cper*HydR^{mat} was first purified with ion-exchange chromatography by passing over 170 mL DEAE resin with 150-250 mM NaCl fractions containing the protein. The solution was further purified by affinity chromatography via Step-Tactin® Superflow® high-capacity resin (IBA) with strep wash buffer (50 mM Tris pH 8.0, 300 mM NaCl, 5% glycerol, and 5 mM sodium dithionite) and strep elution buffer (50 mM Tris pH 8.0, 300 mM NaCl, 5% glycerol, 5 mM sodium dithionite, and 2.5 mM desthiobiotin). Then, the protein was buffer-exchanged into 50 mM Tris pH 8, 300 mM NaCl, 5% glycerol, and concentrated to levels required for individual experiments.

Protein expression and isolation of *Cper*HydR$^{sol/pel}$: In the case of *in-vitro* matured proteins (*Cper*HydR^{sol} and *Cper*HydR^{pel}), *hydR* gene was augmented with a flexible linker follower by the Strep-tag® II sequence at the C-terminal (DIWSVGVKLFGGGSGGGSGGGS**WSHPQFEK**). The gene was synthesized by Twist Bioscience and cloned into a pE-SUMOpro vector conferring kanamycin resistance (LifeSensors) to be expressed as a 6xHis-SUMO-*Cper*HydR fusion construct. This plasmid was then used to transform BL21 (DE3) cells for recombinant expression. Individual colonies were then selected and grown in 5mL LB media cultures supplemented with 50µg/mL kanamycin. A stock culture was made by supplementing a sample of this culture with 25-50% glycerol and stored at -80° C.

The starter culture in 150 mL LB media supplemented with 50 µg/mL kanamycin was grown at 37° C overnight (12 – 18 hours), shaking at 180 RPM (New Brunswick Scientific I 24 incubator shaker). 30 ml of the starter culture was then transferred to 12L of LB media in four 8L polypropylene jugs (Fischer Scientific). The cultures were supplemented with 100 mM MOPS pH 7.4, 2 mM ferric ammonium citrate, 2 mM L-cysteine, and 50µg/mL kanamycin and shaken aerobically at 37° C at 150 RPM (New Brunswick C25 Incubator shaker). At OD_{600} of 0.6 ± 0.2, the cultures were cooled on ice for ≥ 30 minutes, then supplemented with 0.5% glucose and 25 mM sodium fumarate. The cultures were then transferred to 2-liter bottles capped with a septum and shaken at 100 RPM (New Brunswick Excella E25 incubator shaker)

for 30 minutes at 20° C. Then, via a syringe, the cultures were supplemented with 8.4 μg/ml of kanamycin and sodium dithionite (at 2.5-5 mM final concentration) before protein expression was induced with a final concertation of 0.5 mM IPTG at 20 ^{o}C and shaken for 20 hours at 90-110 RPM. Cells were harvested in an anaerobic glovebox (Coy Labs) under 97% N_2 and 3% H_2, pelleted in hermetically sealed 1L centrifuge flasks at 4 ^{o}C at 8,000 x g for 12 minutes (Thermo Fischer Scientific Sorvall^{TM}). Typically, the cell pellet was frozen at -80° C prior to cell lysis.

All sample manipulations were carried out anaerobically under 97% N_2 and 3% H_2 in a Coy Labs glovebox. The cell pellet was suspended in 100 mL of lysis buffer (50mM HEPES pH 7.5, 300 mM KCl, 5 mM imidazole, 2.5 mM sodium dithionite) supplemented with 1 mg/mL lysozyme, 0.1 mg/mL DNaseI, and 0.2 mg/mL PMSF. Cells were lysed by sonication (Sonics & Materials Inc. Model VCX 750) with 2s bursts/8s delay sequence at 70% amplitude on ice for 1 hour. The lysate was then centrifuged at 40,000 x g for 1.5 hours at 4 ^{o}C to separate soluble and insoluble fractions.

The supernatant of cell lysate (containing *Cper*HydR^{sol}) was purified by affinity chromatography using Co-NTA (G-biosciences) resin in a gravity-flow column using standard procedures with wash buffer containing 100mM HEPES pH 7.5, 300mM KCl, 30mM imidazole, 2.5mM sodium dithionite and a similar elution buffer containing 300mM imidazole. For the re-solubilization of the protein from the cell pellet (*Cper*HydR^{pel}), the pellet from centrifugation was added to a 50-100 ml 9M urea, 100 mM HEPES, pH 8.3, 2.5 mM sodium dithionite buffer and stirred overnight at room temperature. To refold *Cper*HydR^{pel}, the solution was diluted with de-ionized water to a 6 M urea solution. Then, the solution was loaded onto a gravity flow column containing Co-NTA (G-biosciences) resin. The column was then washed with a wash buffer (containing no urea) over 4 column volumes (200 mL). The protein was then eluted with the 300mM-imidazole elution buffer.

The eluents of *Cper*HydR^{sol} and *Cper*HydR^{pel} were then rebuffered to a solution of 100mM HEPES pH 7.5, 300mM KCl, and 5 mM dithiothreitol or by dilution/concentration cycles reducing the imidazole concentration below 150 mM required for the next step (final volume <5 ml). Next, the SUMO tag was cleaved with ULP1 protease (LifeSensors) overnight while stirring on ice following manufacturer specifications. The sample was then purified via Step-Tactin® Superflow® high-capacity resin (IBA) with strep wash buffer (100 mM HEPES pH 8.0, 150 mM KCl, 2 mM sodium dithionite) and strep elution buffer (100 mM HEPES pH 8.0, 150 mM KCl, 2 mM sodium dithionite, and 2.5 mM desthiobiotin). The success of each step was verified by SDS-PAGE. Trypsin digest fragments were analyzed by mass spectrometry (PSU Core Facilities) to confirm the identity of the purified proteins.

The [4Fe-4S] clusters were reconstituted using previously established protocols.[3] In short, under strictly anaerobic conditions inside a glovebox (Coy Labs) apo-*Cper*HydR$^{sol/pel}$ was rebuffered into a HEPES/dithiothreitol buffer (100 mM HEPES, pH 7.5, 5 mM dithiothreitol (DTT), 500 mM KCl) using a PD-10 desalting column. Protein was then concentrated to a final concentration of approximately 20 μM. Then, $FeCl_3 \bullet 6H_2O$ was added to a final concentration of 400 μM incrementally over 7.5-15 minutes while the solution was stirred on ice. Next, $Na_2S \bullet 9H_2O$ was added incrementally over 15-30 minutes to a final concentration of 400 μM. This solution was then allowed to stir overnight on ice. The solution was then centrifuged for 30 minutes at 10,000 x g while at 4 ^{o}C (Thermo Fischer Scientific SorvallTM), concentrated and buffer-exchanged using a PD-10 column (GE Healthcare). Both *Cper*HydR^{pel} and *Cper*HydR^{sol} showed roughly the same Fe content per protein of 10.4±0.8 and 10 ± 2 respectively.

The reconstitution of the H-cluster was accomplished by incubation with a synthesized $Fe_2[\mu\text{-}S_2C_2H_4NH](CO)_4(CN)_2$ precursor (complex 1) of the [2Fe] H-subcluster. Complex 1 was synthesized according to previously published protocols.[3–6] Chemical identity of Complex 1 was verified by FT-IR. The [4Fe-4S]-containing *Cper*HydR$^{sol/pel}$ (50 μM) in 100 mM TAPS, pH 8.0, with 300 mM KCl and 5 mM sodium dithionite was incubated with a 5-fold excess of complex 1 overnight while stirred at 4 ^{o}C. Excess complex 1 and sodium dithionite were removed with a PD-10 column, and the protein was stored in a storage buffer (100 mM HEPES pH 7.5, 300 mM KCl, and 10% glycerol (v/v)). Prior to any further experimentation, the

protein was buffer-exchanged to experiment-specific buffers using a PD-10 column (GE Healthcare) and incubated for 1 hour in a 100% H_2 atmosphere. Additional purification via size-exclusion chromatography was performed with an S-200 HR (Cytiva) column on Akta Go FPLC (Cytiva) at room temperature. For these experiments, the fully activated protein sample was buffer exchanged into 100 mM MOPS, pH 7.5, 150 mM KCl; the flow rate was set to 0.5 ml/min. Our elemental analysis noted the presence of 3-4 ± 1 Zn ions per protein likely retained at rubredoxin and the diiron sites, which are known to incorporate zinc during recombinant expression.[7–9]

Expression, and isolation of truncated *Cper*HydR$^{\Delta N}$: The gene of the *Cper*HydR$^{\Delta N}$ was obtained from the amino acid sequence of the wild-type *Cper*HydR by removing the first 438 amino acids comprising the HydA domain. The resulting sequence was then codon-optimized for *E. coli* expression, augmented with an N-terminal 6xHis tag, and a C-terminal Strep-tag® II sequence (SA**WSHPQFEK**), synthesized by Twist Biosciences and cloned into the pET28a(+) vector (conferring kanamycin resistance) using *BamHI* and *XhoI* restriction sites. This vector was then used to transform *E.coli* BL21 (DE3) cells for recombinant expression. Individual colonies were then selected and grown in 5mL LB media cultures supplemented with 50µg/mL kanamycin. A stock culture was made by supplementing a sample of this culture with 25-50% glycerol and stored in -80° C freezer. This stock culture was used to grow 12-liter cultures with aerobic expression and purification procedures similar to those used for *Cper*HydR^{sol}, except for adding sodium dithionite during the expression and performing SUMO-tag removal.

H_2 Activity assays. H_2 oxidation activity assays for *Cper*HydR$^{mat/sol}$ were carried out by measuring the reduction of methylene blue (MB) by UV-Vis. For *Cper*HydR^{mat}, the reaction buffer was 50 mM Tris pH 8.0, 200 mM NaCl, 5% glycerol, and 50 µM MB. For *Cper*HydA^{sol}, the reaction buffer was 100 mM MOPS, pH 7.5, 300 mM KCl, 82 µM MB. The reaction cuvette's headspace was exchanged with 100% H_2 at 1 atm by repeated vacuum/H_2 cycles on a Schlenk line before the experiment. The activity assay reaction was initiated either by the addition of methylene blue from a stock solution (0.5 mM) or by the addition of *Cper*HydR$^{mat/sol}$ (0.08- 0.2 µg), with a final reaction volume of 0.5 mL inside a sealed anaerobic UV cuvette (Starna Cells). Measurements were carried out at room temperature using either Carry 60 or Cary 4000 UV-Vis spectrometer (Agilent Technologies). The solution was constantly stirred during the measurements with a miniature magnetic stir bar. The absorbance at 613 nm was monitored over the course of several minutes until no changes in absorbance were observed. The data was fit with an exponential decay function, and specific activity for H_2 uptake by *Cper*HydR was calculated using a measured extinction coefficient of MB (ε_{613}= 11,300 $M^{-1}cm^{-1}$).

H_2 production activities of *Cper*HydR^{mat} were assayed from reduced methyl viologen. Reaction volumes of 2 mL were carried out in 13 mL Wheaton septa sealed vials containing 5 mM methyl viologen, 10 mM sodium dithionite, 50 mM Tris pH 8, 300 mM NaCl, 5% glycerol, and 3 to 50 µg of enzyme per assay. Reactions were initiated by the addition of methyl viologen and were carried out at 37° C for 10 to 30 minutes. Hydrogen production was measured by gas chromatography (Agilent Technologies).

H_2O_2 reduction by *Cper*HydR$^{\Delta N}$. The reduction of hydrogen peroxide was measured in a chronoamperometric experiment in a home-built electrochemical cell. Per Clark et al.,[10] the H_2O_2-sensing needle microelectrode consists of an 18-gauge hypodermic stainless-steel needle as a reference/counter electrode and PFA-coated Pt wire (0.2mm OD, A-M Systems) fed through the needle as a working electrode (PFA coating was stripped appropriately) poised at +800 mV. The cell was a 1.5 ml vial (Eppendorf) with a miniature magnetic stirrer. The stock protein solution in 200 mM HEPES, pH 7.0, and 200 mM KCl was incubated with a five-fold excess of dithiothreitol (DTT), turning the solution from red to green upon the addition of DTT. The protein solution was then buffer-exchanged via PD-10 column to remove excess DTT and concentrated to the final concentration of *Cper*HydR$^{\Delta N}$ of 1.85 mM. The current-H_2O_2 concentration relationship was established by the repeated addition of 0.3 µL of 1030 mM H_2O_2 to the 600 µL of buffer solution (200 mM HEPES, pH 7.0, and 200 mM KCl) prior to protein addition. Then, 50 µL aliquots of the

protein stock solution were added until the complete reduction of the H_2O_2 electrochemical signal. The concentration of the H_2O_2 stock solution was calibrated by quantifying the oxidation of reduced benzyl viologen, which was monitored by bleaching the 560 nm band.

H_2O_2 reduction by *holo-Cper*HydR^{sol}. The H_2O_2 reduction activity was carried out by measuring the oxidation of reduced benzyl viologen (BV) in 100 mM MOPS, pH 7.5, 150 mM KCl, 128 µM BV. Activated *Cper*HydR was incubated with 1.4 mM H_2O_2 (in 100 mM MOPS, pH 7.5, 150 mM KCl) in a sealed gas-chromatography vial. The headspace of the vial was pressurized with 100% H_2 gas. 100 µL aliquots were collected at varied time points, and the protein was separated from the H_2O_2 via a 30kDa Amicon filter (Sigma-Aldrich) to avoid potential side reactions of protein with BV. 25 µL of the flow through was injected into a sealed UV/Vis cuvette containing the one-electron reduced $BV^{\bullet +}$. The oxidation of $BV^{\bullet +}$ by H_2O_2 results in bleaching of absorbance at 560 nm (ε_{560} = 7800 M^{-1} cm^{-1}), which is proportional to peroxide concentration (2:1 ratio $BV^{\bullet +}$:H_2O_2). Each activity measurement was repeated 3-5 times to afford error analysis. All data processing was performed using the Kazan Viewer data processing suite in MATLAB™.[11]

ICP-AES. Metal analysis of the apo and holo-*Cper*HydR was achieved by dissolution of metals in 2-5% nitric acid and separation from the protein debris by centrifugation. Analysis was completed by ICP-AES (PSU Core Facilities).

Protein film electrochemistry. All PFV experiments were carried out inside an anaerobic glove box (Coy Labs) using a WaveNow Wireless Potentiostat (Pine Research) connected to a rotator (Pine Research). The working rotating disc electrode (RDE) is built of a 5mm OD edge-plane disc of pyrolytic graphite PG-HT (GraphiteStore) bonded to a stainless-steel threaded insert using heat-cured Silver Conductive epoxy (Silver-Bond 4, Epoxy international). The assembly was encased in 12 mm OD 3D printed housing using Siraya Tech Sculpt resin and bonded using non-conductive epoxy. The electrode surface was polished down to 1200 grit and sonicated in de-ionized water. The surface of the electrode was conditioned by several cyclic voltammetry scans between 0 mV and -800 mV vs NHE at 100 mV/s. The reference electrode was a single junction Ag/AgCl reference electrode (BASI). The counter electrode was a polymer-encased gold rod (BASI). The reference electrode was calibrated using methyl viologen and 150 mM KCl, 100 mM HEPES buffer solution at pH 7.0 ($E_0(MV^{2+}/MV^{\cdot +})$ = -445 mV vs NHE). To generate protein films, *Cper*HydR was equilibrated in 100 mM HEPES, pH 6.5 - 7.5, 150 mM KCl, and diluted to approximately 20 µM. A 15 µL aliquot of this solution was then placed onto the graphite electrode and incubated at room temperature for 15 minutes. The excess solution was then removed from the surface of the electrode prior to being installed into the cell. All PFV experiments were carried out in the following buffers: 100 mM of MES (pH6), HEPES (pH7), TAPS (pH8), CHES (pH9), and 150 mM of KCl. The pH of the solution was adjusted using concentrated HCl and 50% NaOH as needed. Unless otherwise specified, the buffer solution was purged with 100% H_2 at 100 SCCM controlled by a mass flow controller (Cole-Parmer). The rotation speeds of the RDE were set to 1000 rpm.

FTIR measurements. FTIR spectra were collected either using a Nicolet 6700 or Nicolet is50 FTIR Spectrometer (Thermo Fisher Scientific). Both instruments are equipped with CaF_2 beam splitter and a liquid-nitrogen-cooled mercury cadmium telluride (MCT) detector. The sample cell consisted of a set of CaF_2 windows with either a PTFE-based spacer with a path length of 10-40 µm or a 15 µm copper spacer with a custom-built air-tight holder. Unless otherwise specified, the data was acquired at 2 cm^{-1} resolution.

EPR measurements. EPR spectra were collected on either an X-band Bruker E500 spectrometer equipped with an SHQ resonator or a Magnettech MS5000X spectrometer. The Bruker E500 instrument was equipped with a cryogen-fee helium system (ColdEdge Technologies) and MercuryITC temperature controller (Oxford Instruments). The MS5000X instrument was equipped with a continuous-flow LHe cryostat Oxford Instruments ESR900 and LakeShore 335 temperature controller. Samples were prepared anaerobically inside a glovebox and placed into clear fused quartz tubes with 4mm O.D./3mm I.D. (QSI

quartz). Tubes were sealed with either septa or tape, taken outside the glovebox, and frozen directly in liquid nitrogen. Unless specified otherwise, EPR spectra were acquired at 1mW microwave power, 100kHz modulation amplitude, and 1 mT modulation amplitude. Spectral simulations were performed in MatLab using Kazan Viewer[11] and utilizing *'pepper'* function from EasySpin 6.0[12].

Figure preparation. All Figures were prepared with Adobe Illustrator using the outputs of other software. Chemical structures were drawn with ChemDraw (PerkinElmer). The atomic models were generated with UCSF ChimeraX.[13] Data plots were generated directly from the original spectroscopic and electrochemical datasets in MatLab using Kazan Viewer.[11]

Table S1: Comparison of IR frequencies of various relevant experimentally observed states of the H-cluster.

State	Species	Terminal CN^-	Terminal CO	Bridging CO	Source
H_{ox}	***Cper*HydR**	**2086, 2081**	**1974, 1945**	**1745**	**this work**
	*Cp*II	*2082, 2069*	1969, 1945	1752	[14]
	*Cp*I	2082, 2072	1971, 1948	1802	[15]
	*Cb*HydA1	2092, 2081	1964, 1941	1800	[3]
	*Dd*HydAB	2094, 2079	1965, 1940	1802	[16]
	CrHydA1	2088, 2072	1964, 1940	1800	[17]
$H_{ox}CO$	***Cper*HydR**	**2092, 2086**	**2024, 1982, 1959**	**1745**	**this work**
	*Cp*II	*2090, 2074*	*2023, 1975, 1960*	*1752*	[18]
	*Cp*I	2095, 2077	2017, 1974, 1971	1810	[15]
	*Cb*HydA1	2093, 2088	2014, 1970, 1962	*1806*	[3]
	*Dd*HydAB	2096, 2088	2016, 1971, 1963	1810	[16]
	CrHydA1	2092, 2084	2013, 1972, 1964	1810	[17]
$H_{red}(H^+)$	***Cper*HydR**	**2065, 2035**	**1923, (1903), 1896, *1883**	**n.r.**	**this work**
	CpII	*2048, 2034*	1923, (1918), 1889	n.r.	[18]
	CpI	*2082, 2053*	1962, 1915, 1899	n.r.	[15]
	CbHydA1	*2075, 2041*	n.r, 1916, 1893	n.r.	[3]
	DdHydAB	*2079, 2041*	1965, 1916, 1894	*1810*	[16,19]
	CrHydA1	*2083, 2070*	1935, 1891	*1793*	[17]

* – additional minor band that may be due to a different state, such as H_{sred} or $H_{sred}H^+$.
n.r. – not resolved

Table S2. Parameters of EPR simulations in **Figure 3**.

Component	$g_{1,2,3}$	g-Strain(1,2,3)	Linewidth*	Double integral fraction			
				Fig. 3A	Fig. 3B	Fig. 3C	Fig. 3D
H_{ox}	2.0900, 2.0350, 2.0003	0.0110, 0.0058, 0.0090	0.62, 0.0	0.0	0.0	0.24	0.0
H_{ox}-CO	2.0298, 2.0161, 1.9975	0.0077, 0.0057, 0.0026	1.0, 0.28 (1.6, 0.338)**	0.0	0.12	0.27	0.0
$[4Fe\text{-}4S]^{+1}$	2.0638, 1.9466, 1.8918	0.0088, 0.0110, 0.0187	1.38, 0.49	0.68	0.78	0.41	0.0
$[4Fe\text{-}4S]^{+1}$	2.0698, 1.9516, 1.8888	0.0116, 0.0119, 0.0174	2.22, 0.96	0.32	0.0	0.0	0.0
Rbr, di-iron	1.9640, 1.7350, 1.6640	0.0000, 0.0366, 0.0436	3.48, 1.00	0.0	0.0	0.0	1.0
[3Fe-4S]	2.0101, 2.0020, 1.9740	0.0070, 0.0165, 0.0581	0.59, 0.00	0.0	0.10	0.08	0.0

*simulation assumes pseudo-Voigt broadening. The two numbers represent Gaussian and Lorentzian linewidth parameters

** The H_{ox}-CO signal was found to be noticeably broader at higher temperatures due to faster relaxation. Therefore, the simulation of data at 40K (numbers in brackets) assumed a higher broadening.

Table S3. g-values of mixed-valent Fe(II)-Fe(III) centers from various systems.

System	g_1	g_2	g_3	ref
sMMO, as prepared	1.95	1.86	1.77	[20,21]
sMMO, Me_2SO	1.95	1.86	1.77	[21]
sMMO, methanol	1.96	1.84	1.73	[21]
sMMO, with access Me_2SO	1.86	1.77	1.62	[21]
Ferritin*, Synechococcus CC9311*	1.943	1.800	1.760	[22]
Hemerythrin-like E3 ubiquitin ligase	1.95	1.80	1.67	[23]
Hemerythrin*, Phascolopsis gouldii*	1.95	1.86	1.62	[23]
Rubrerythin, *Desulfovibrio vulgaris*	1.98	1.76	1.57	[24]
***Cper*HydR**	**1.964**	**1.735**	**1.664**	**this work**

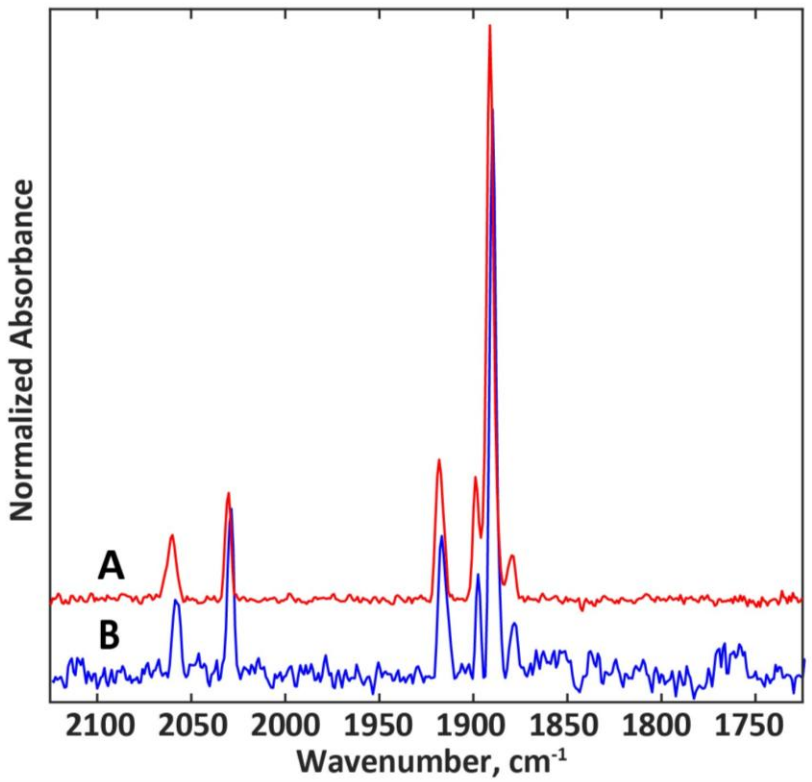

Figure S1: Room temperature FTIR spectra of *Cper*HydR^{mat}. A) As prepared sample. B) Sample treated with 20mM NaDT. The absorbance was normalized to the highest signal amplitude.

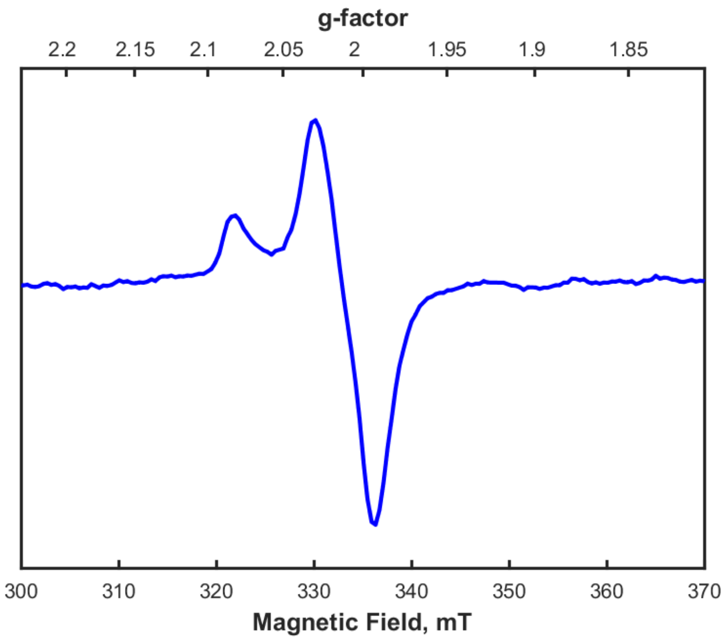

Figure S2: X-band EPR spectrum of H_2O_2-exposed holo-*Cper*HydR^{mat}. Experimental conditions: temperature, 40K; microwave frequency, 9.37786 GHz; microwave power, 1.003 mW.

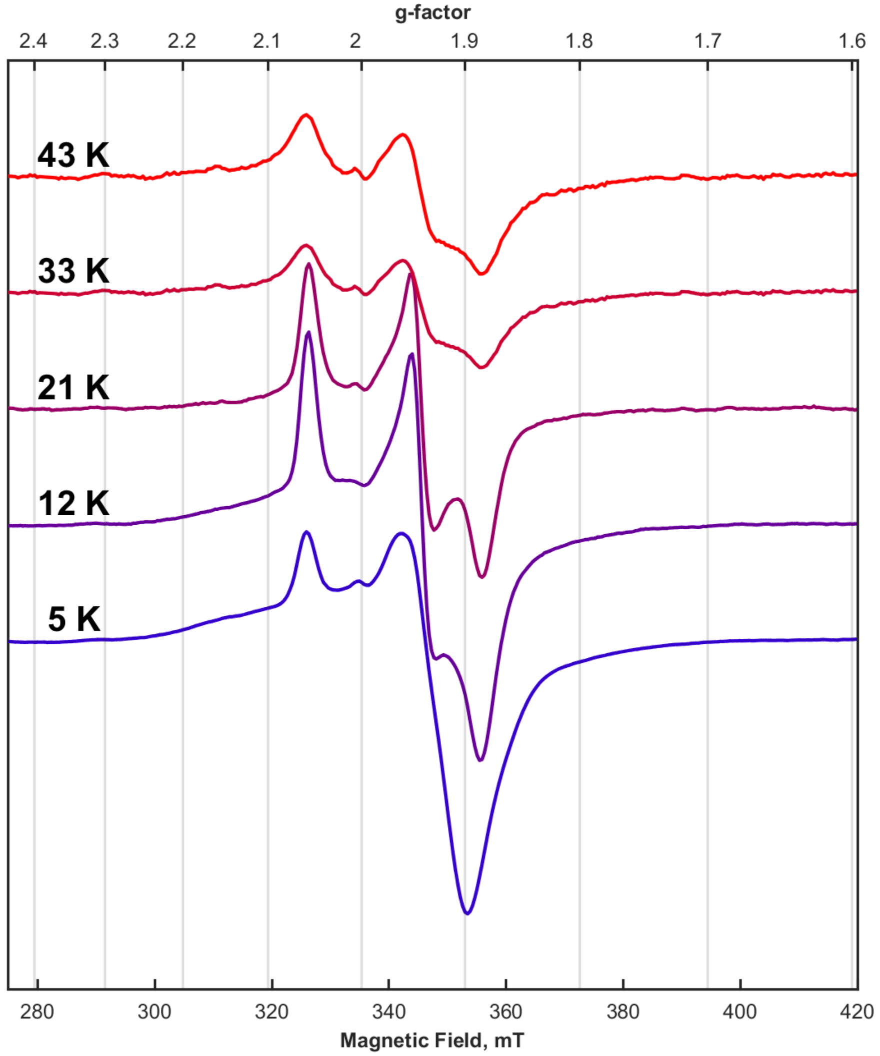

Figure S3. Temperature dependence of X-band EPR spectra of NaDT-treated holo-*Cper*HydR^{mat} at 9.383-9.385 GHz and 1.003 mW. The NaDT reduced sample was prepared in 50 mM Tris pH 8, 200 mM NaCl, 5% glycerol, 10 mM NaDT, 280 uM protein concentration. Numbers above each trace indicate the temperatures at which the spectra were taken. Traces were scaled according to the Curie law for clarity.

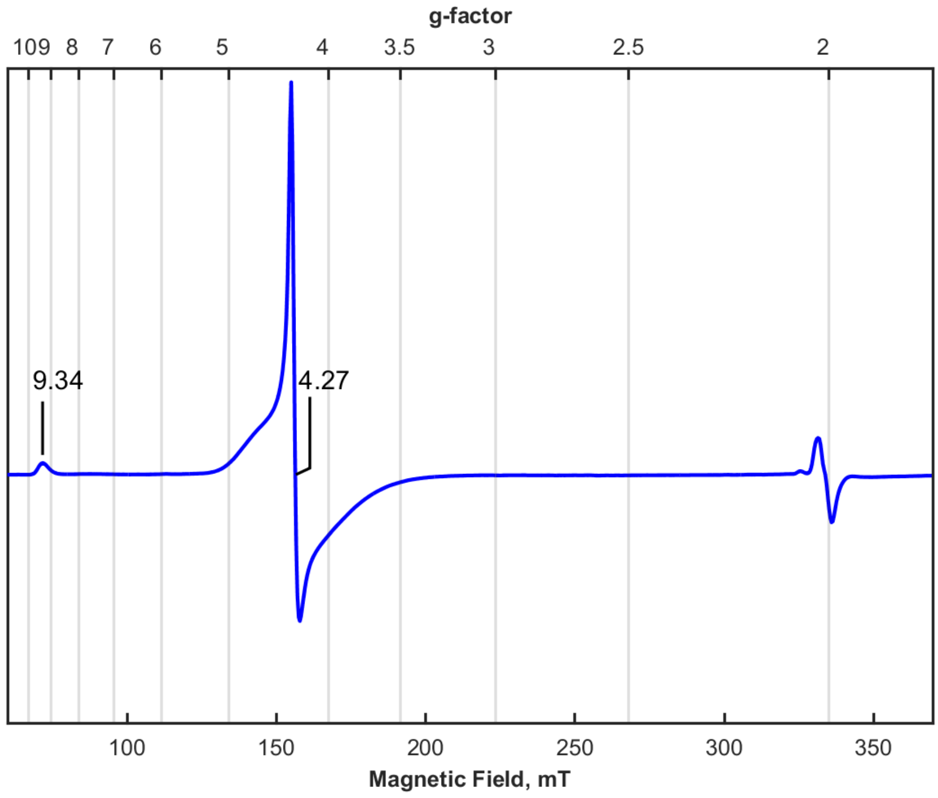

Figure S4. X-band CW EPR spectra of *Cper*HydR^{mat} showing the ferric rubredoxin. Experimental conditions: temperature, 5K; microwave frequency, 9.378071 GHz; microwave power, 1.003 mW.

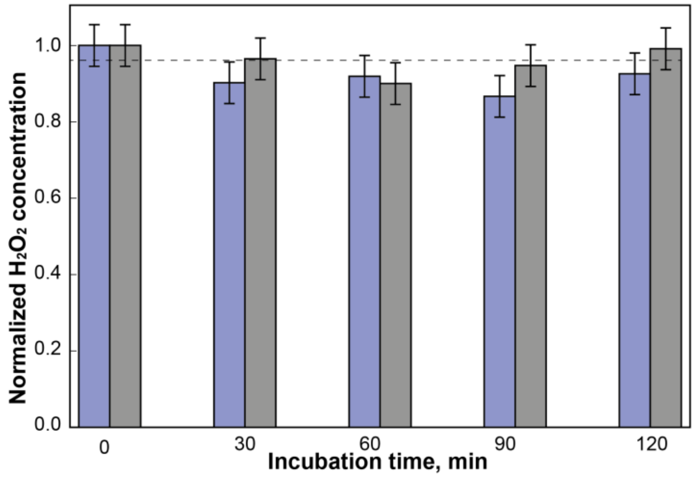

Figure S5. Lack of H_2O_2 reduction by 2 µM *Cper*HydR^{pel} under 1 atm CO (blue bars). Data was accumulated in the presence of 1.3 mM of H_2O_2. Gray bars represent control experiments without protein. The dashed line represents the average value from the control experiments. Data were normalized to the first point taken immediately after initiating the reaction.

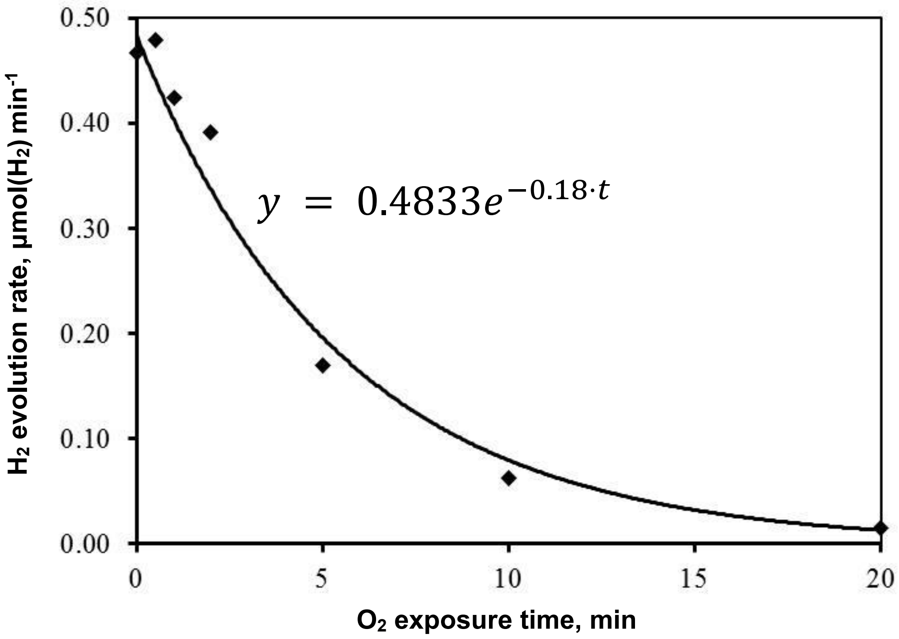

Figure S6. H_2 evolution rate of *Cper*HydR^{mat} upon exposure to air. The equation used to produce solid line is presented above.

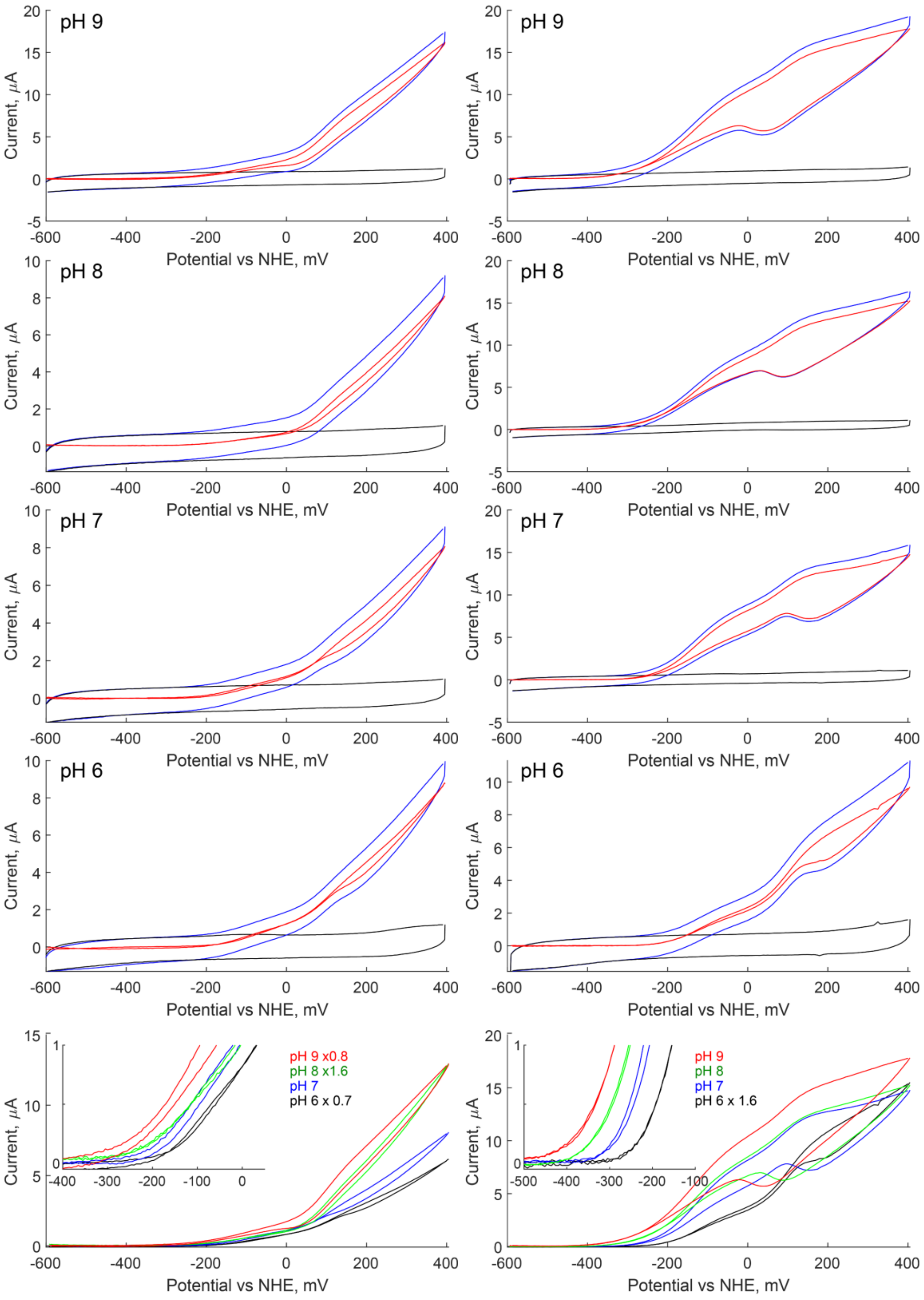

Figure S7. Top four rows: original cyclic voltammetry trances of holo-*Cper*HydR^{sol} (left) and holo-*Cper*HydR^{pel} (right) modified rotating pyrolytic graphite electrode (PGE) as a working electrode measured at pH values of pH 6 (MES buffer), pH 7 (MOPS buffer), pH 8 (TAPS buffer), pH 9 (CHES buffer) as indicated on respective panels. Black traces are baseline measurements, blue traces are the recorded voltammograms, and red traces are the results of subtraction. The bottom two panels show an overlay of baseline subtracted traces. Experimental conditions: room temperature; scan rate, 20 mV/s; rotation speed, 1000 rpm.

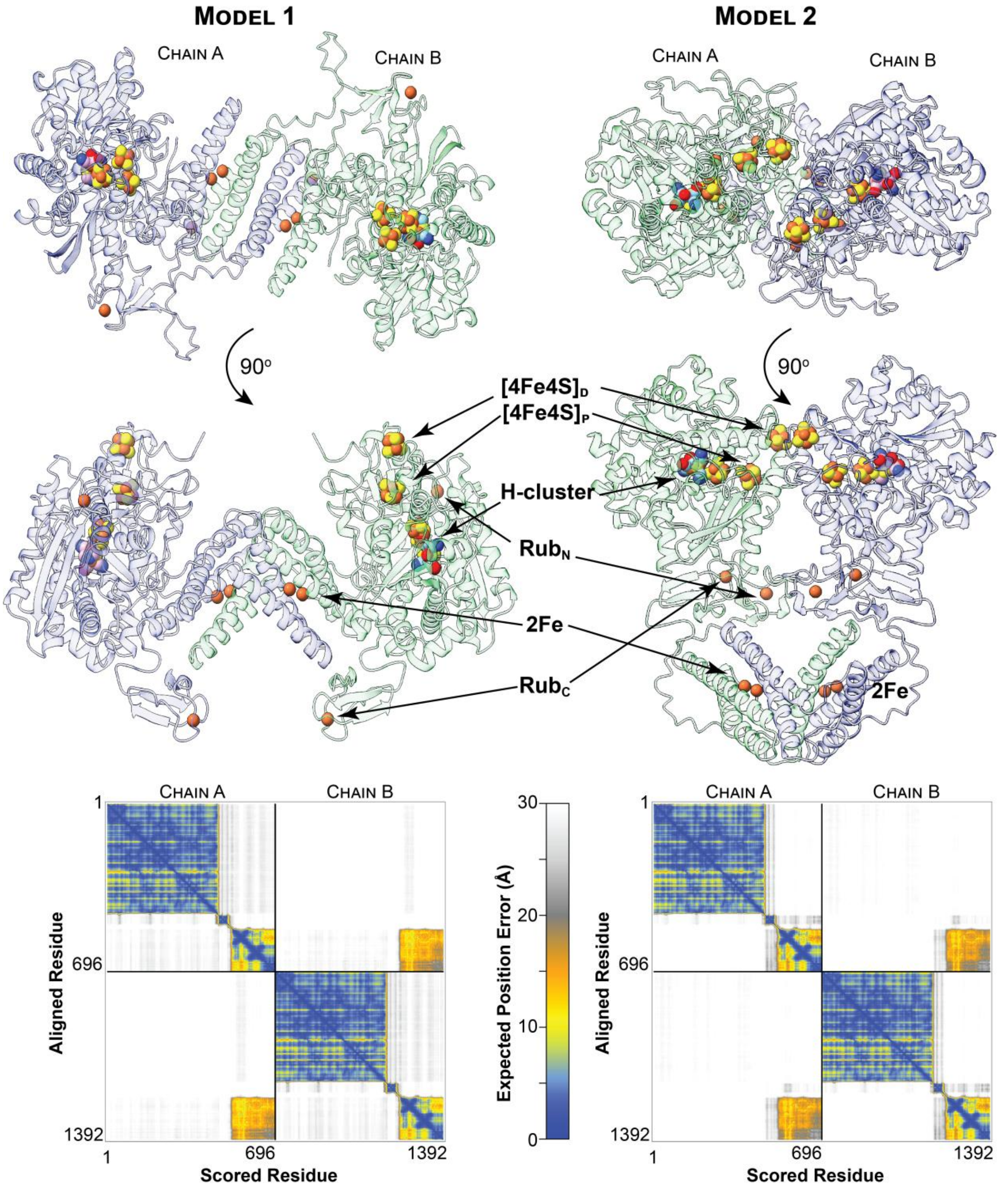

Figure S8. Two highest-scored AlphaFold 3[25] models of *Cper*HydR dimer (left and right) and respective predicted aligned error maps (bottom panels). Colored spheres represent Fe (orange), S (yellow), O (red), N (blue) of metallocofactors positioned according to the alignment of predicted structures with relevant subdomains of *Cp*I (PDB: 6N59) and *Pf*Rbr(2HR5) crystallographic data. The predicted template modeling (pTM) and the interface predicted template modeling (ipTM) scores:

Model 1 pTM=0.41, ipTM=0.30, ranking score=0.34

Model 2 pTM=0.41, ipTM=0.28, ranking score=0.32

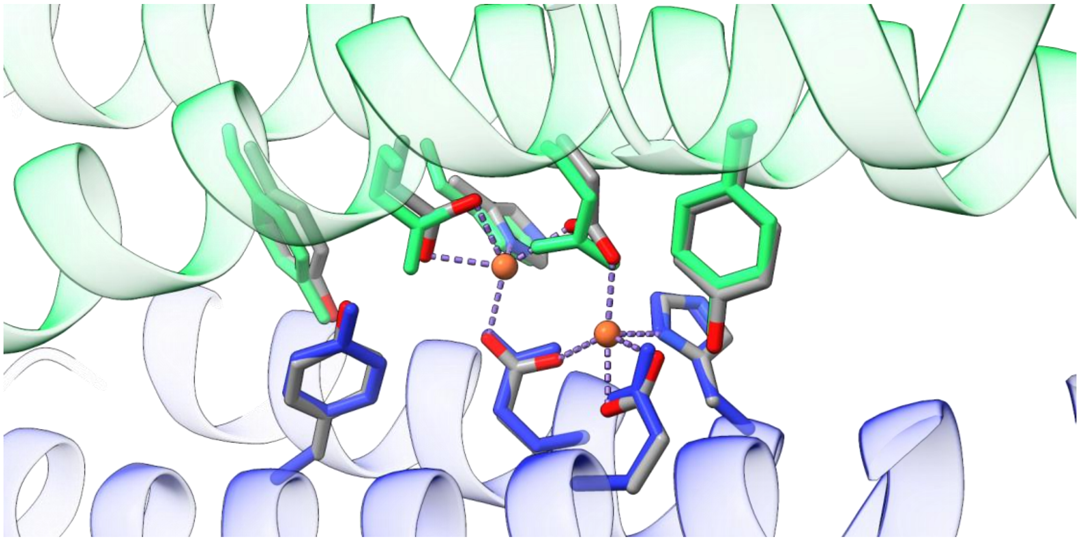

Figure S9. The predicted binding site of the diiron cofactor of *Cper*HydR (dark blue and green residues, model 1) as compared to the positioning of the corresponding residues in the *Pf*Rbr crystallographic structure (PDB 2HR5, displayed residue atoms colored by elements: gray – C, red – O, blue – N, orange – Fe). This comparison indicates the plausibility of the existence of a diiron cofactor in dimeric *Cper*HydR.

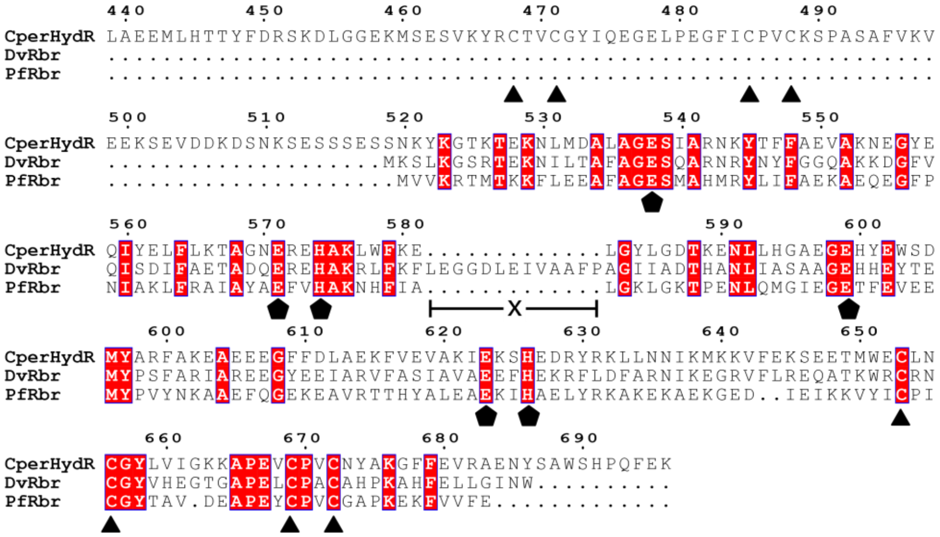

Figure S10. Sequence alignment of various Rbr domains with the relevant portion of the *Cper*HydR sequence. "X" indicates 13 amino acids that proposedly prevent four-helix bundling in a monomer. Triangles indicate Fe-coordinating cysteines in rubredoxin-like domains. Pentagons indicate residues coordinating the diiron center. *Dv*Rbr - *Desulfovibrio vulgaris Rbr*, sequence extracted from PDB 1RYT. *Pf*Rbr - *Pyrococcus furiosus* Rbr*,* sequence extracted from PDB 2HR5.

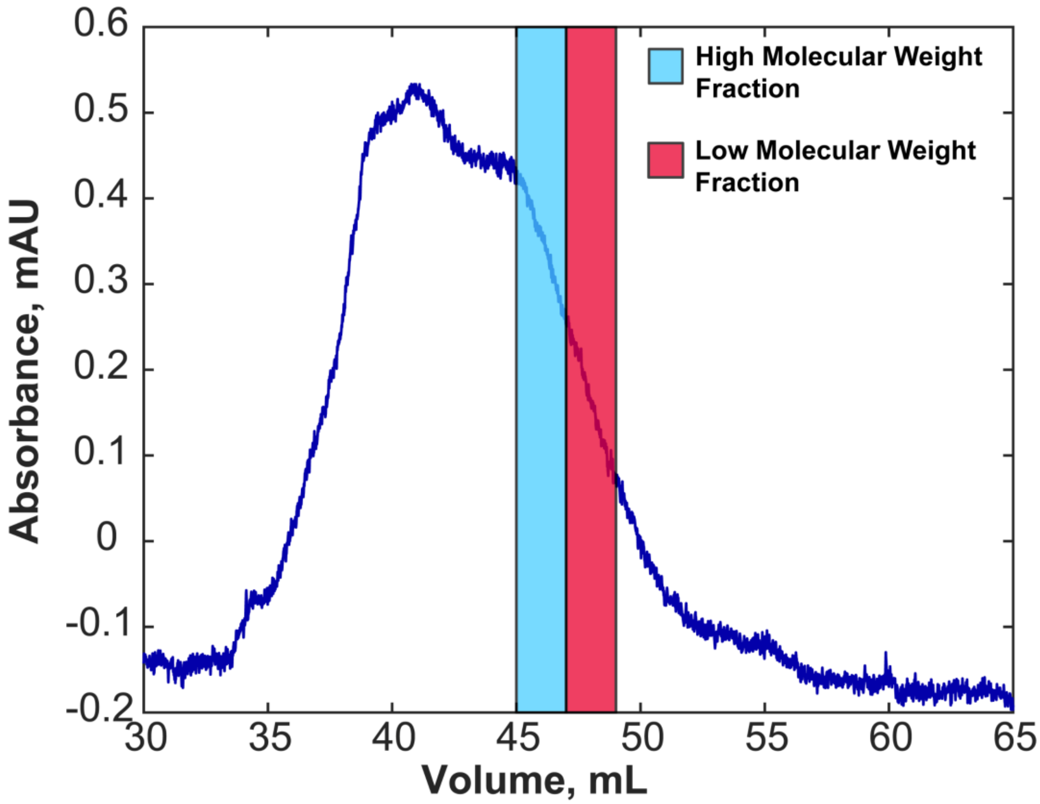

Figure S11. Size exclusion chromatography of *Cper*HydR^{pel} (blue). Red and Blue areas indicate the fractions collected for protein film voltammetry analysis as low molecular weight (red) and high molecular weight (blue) fractions. Experimental conditions, flow rate, 0.5 ml/min, buffer, 100 mM MOPS, pH 7.5, 150 mM KCl.

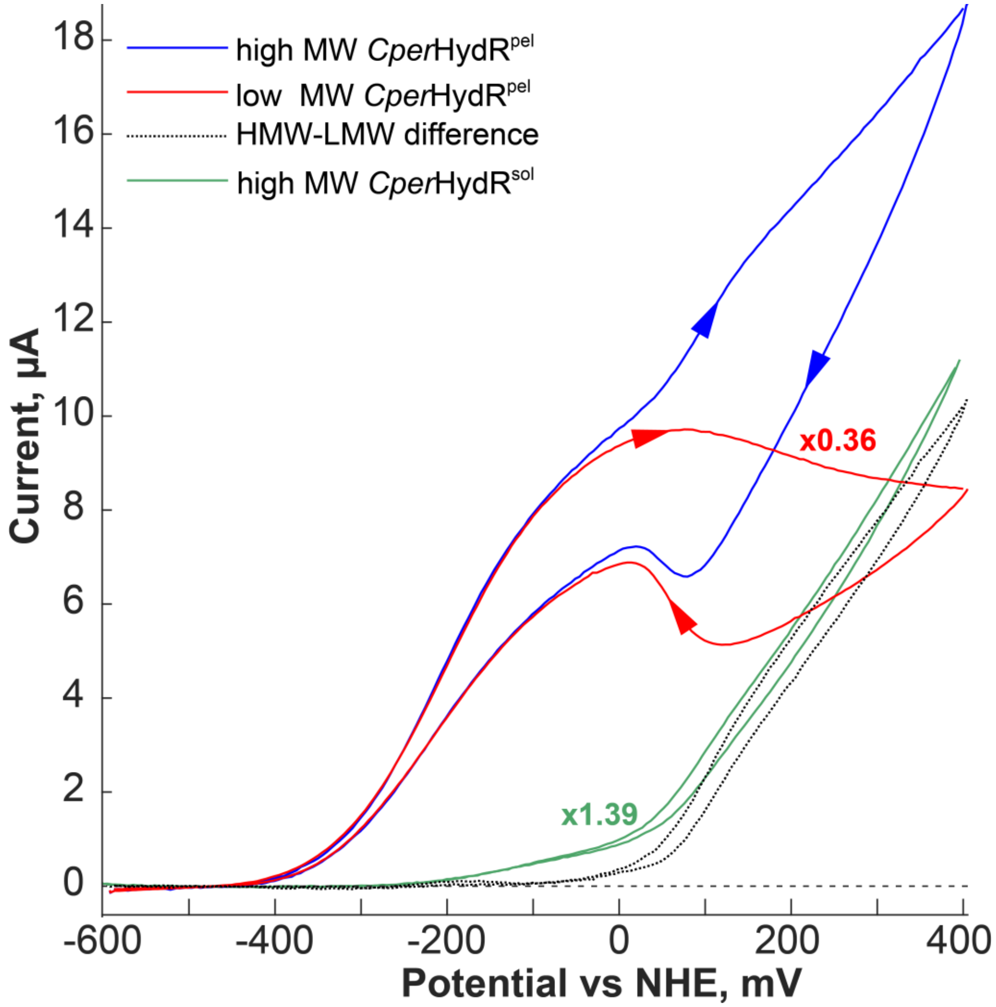

Figure. S12. Comparison of PFV traces of low molecular weight (red) and high molecular weight (blue) fractions of *Cper*HydRpel taken at pH 8. The dashed black line represents the difference between red and blue traces. The green line is the *Cper*HydRsol trace (same as the blue trace in Fig. 6C). There is a distinct difference between the two traces in the E > 0 mV region, while traces are nearly identical at the E < 0 mV when normalized by current at -200 mV. The difference between the two traces (see black trace in **Fig. 11**) is remarkably similar to that of *Cper*HydR^{sol} obtained under the same pH (see black trace in **Fig. 11**). This experiment strongly suggests that the CV trace of the LMW fraction is representative of a monomer and likely characteristic of the HydA domain in the absence of the fully assembled di-iron site of rubrerythrin. Then, the difference trace, which is the major component of *Cper*HydR^{sol} in **Fig. 11**, represents the catalytic current of the homodimer with all identified metallocofactors present.

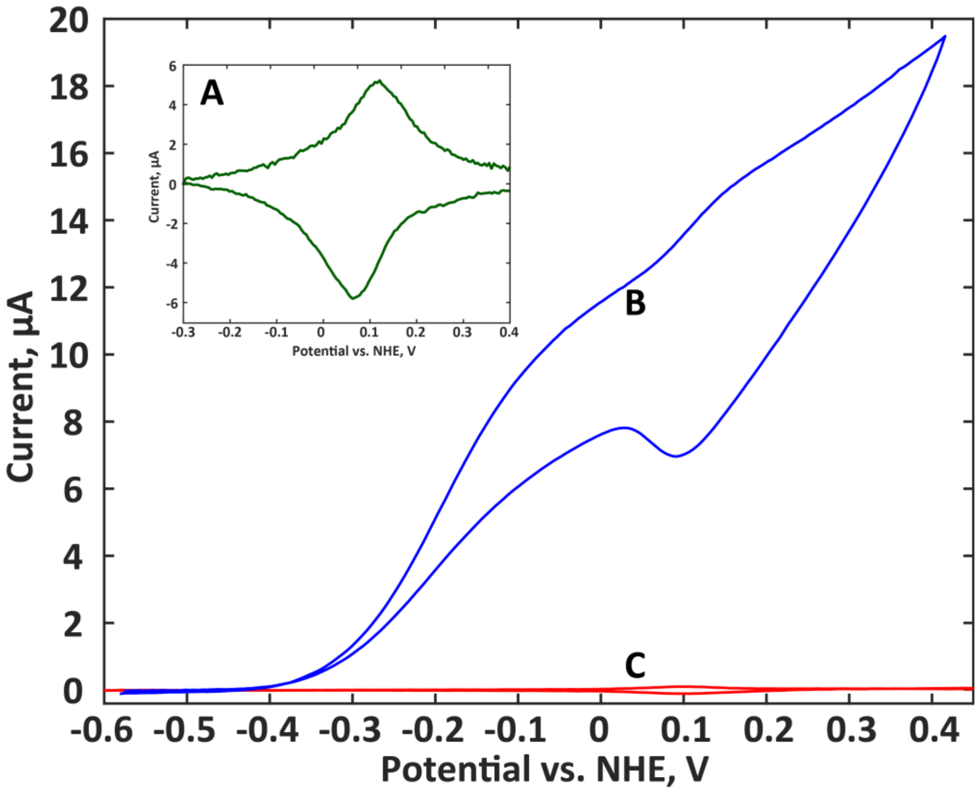

Figure S13. Protein film cyclic voltammetry (CV) of *Cper*HydR$^{\Delta N}$ (A, C) and *Cper*HydR^{pel} High molecular weight (B) modified pyrolytic graphite electrode measured at room temperature. A) CV of *Cper*HydR$^{\Delta N}$ measured at 1000 mV/s scan rate. B) CV of *Cper*HydR^{pel} measured at 20 mV/s scan rate. C) CV of *Cper*HydR$^{\Delta N}$ measured at 20 mV/s scan rate. Experimental conditions: buffer, 100 mM HEPES, pH 8.00, 150 mM KCl; rotating speed, 1000 rpm; electrolyte solution was bubbled with H_2 gas at 100 sccm flow rate.

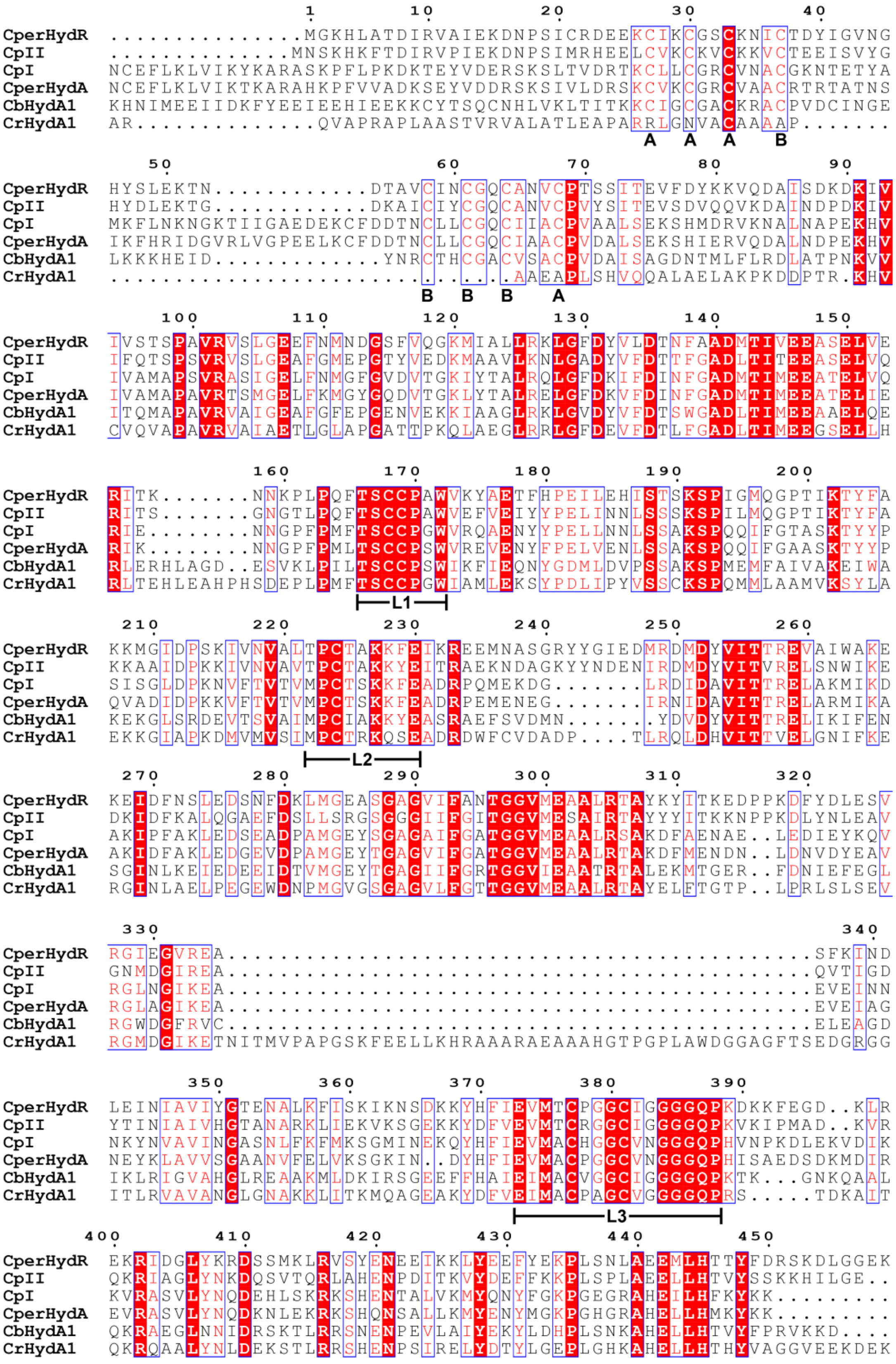

Figure S14. A portion of the sequence alignment of various [FeFe] hydrogenases. L1, L2 and L3 are regions of the sequence containing the conserved H-cluster binding motifs (see main text). A and B indicate cysteines coordinating the two accessories [4Fe-4S] clusters present in *Cper*HydR, distal and proximal to the H-cluster, respectively.

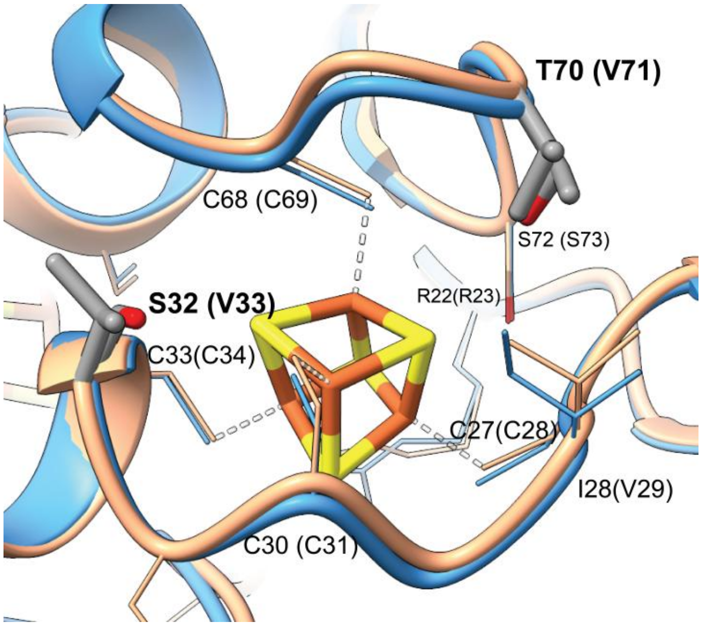

Figure S15. Comparison of the amino acid environment of the distal [4Fe-4S] cluster in *Cper*HydR and *Cp*II. Structures were generated by AlphaFold 3; see model 1 from Figure S8 for *Cper*HydR. Positioning of the FeS cluster was modeled based on the structure of *Cp*I (PDB: 6N59). Light blue ribbons represent *Cper*HydR, and tan ribbons designate *Cp*II. The amino acid position of relevant residues for *Cper*HydR are indicated; residues in parenthesis are the equivalent positions for *Cp*II. See sequence alignment in Figure S14 for details.

References:

1. King, P. W., Posewitz, M. C., Ghirardi, M. L. & Seibert, M. Functional Studies of [FeFe] Hydrogenase Maturation in an *Escherichia coli* Biosynthetic System. *J Bacteriol* **188**, 2163–2172 (2006).
2. Mulder, D. W. *et al.* Investigations on the Role of Proton-Coupled Electron Transfer in Hydrogen Activation by [FeFe]-Hydrogenase. *J. Am. Chem. Soc.* **136**, 15394–15402 (2014).
3. Corrigan, P. S., Tirsch, J. L. & Silakov, A. Investigation of the Unusual Ability of the [FeFe] Hydrogenase from *Clostridium beijerinckii* to Access an O_2-Protected State. *J. Am. Chem. Soc.* **142**, 12409–12419 (2020).
4. Seyferth, D., Henderson, R. S. & Song, L. C. Chemistry of .mu.-dithio-bis(tricarbonyliron), a mimic of organic disulfides. 1. Formation of di-.mu.-thiolate-bis(tricarbonyliron) dianion. *Organometallics* **1**, 125–133 (1982).
5. Li, H. & Rauchfuss, T. B. Iron Carbonyl Sulfides, Formaldehyde, and Amines Condense To Give the Proposed Azadithiolate Cofactor of the Fe-Only Hydrogenases. *J. Am. Chem. Soc.* **124**, 726–727 (2002).
6. Seyferth, Dietmar., Womack, G. B., Henderson, R. S., Cowie, Martin. & Hames, B. W. Michael-type addition reactions of bis(.mu.-mercapto)bis(tricarbonyliron): proximity-induced formation of bidentate organosulfur ligands. *Organometallics* **5**, 1568–1575 (1986).
7. Jin, S., Kurtz, D. M., Liu, Z.-J., Rose, J. & Wang, B.-C. Displacement of iron by zinc at the diiron site of Desulfovibrio vulgaris rubrerythrin: X-ray crystal structure and anomalous scattering analysis. *Journal of Inorganic Biochemistry* **98**, 786–796 (2004).
8. Weinberg, M. V., Jenney, F. E., Cui, X. & Adams, M. W. W. Rubrerythrin from the Hyperthermophilic Archaeon *Pyrococcus furiosus* Is a Rubredoxin-Dependent, Iron-Containing Peroxidase. *J Bacteriol* **186**, 7888–7895 (2004).

9. Jin, S., Kurtz, D. M., Liu, Z.-J., Rose, J. & Wang, B.-C. X-ray Crystal Structure of *Desulfovibrio vulgaris* Rubrerythrin with Zinc Substituted into the [Fe(SCys) $_4$] Site and Alternative Diiron Site Structures ᐟ. *Biochemistry* **43**, 3204–3213 (2004).
10. Clark, C. M., Ruszala, B. M. & Ehrensberger, M. T. Development of durable microelectrodes for the detection of hydrogen peroxide and pH. *Med Devices & Sens* **3**, e10074 (2020).
11. Silakov, A. & Epel, B. Software: Kazan Viewer https://github.com/AlexeySilakov/KazanViewer. (2024).
12. Stoll, S. & Schweiger, A. EasySpin, a comprehensive software package for spectral simulation and analysis in EPR. *Journal of Magnetic Resonance* **178**, 42–55 (2006).
13. Meng, E. C. *et al.* UCSF ChimeraX : Tools for structure building and analysis. *Protein Science* **32**, e4792 (2023).
14. Artz, J. H. *et al.* Tuning Catalytic Bias of Hydrogen Gas Producing Hydrogenases. *J. Am. Chem. Soc.* **142**, 1227–1235 (2020).
15. Chen, Z. *et al.* Infrared Studies of the CO-Inhibited Form of the Fe-Only Hydrogenase from *Clostridium pasteurianum* I: Examination of Its Light Sensitivity at Cryogenic Temperatures. *Biochemistry* **41**, 2036–2043 (2002).
16. Roseboom, W., De Lacey, A. L., Fernandez, V. M., Hatchikian, E. C. & Albracht, S. P. J. The active site of the [FeFe]-hydrogenase from *Desulfovibrio desulfuricans*. II. Redox properties, light sensitivity and CO-ligand exchange as observed by infrared spectroscopy. *J Biol Inorg Chem* **11**, 102–118 (2006).
17. Adamska, A. *et al.* Identification and Characterization of the "Super-Reduced" State of the H-Cluster in [FeFe] Hydrogenase: A New Building Block for the Catalytic Cycle? *Angew Chem Int Ed* **51**, 11458–11462 (2012).
18. Artz, J., Mulder, D., Ratzloff, M., Peters, J. & King, P. The Hydricity and Reactivity Relationship in [FeFe]-hydrogenases. Preprint at https://doi.org/10.21203/rs.3.rs-77874/v1 (2020).
19. Birrell, J. A. *et al.* Spectroscopic and Computational Evidence that [FeFe] Hydrogenases Operate Exclusively with CO-Bridged Intermediates. *J. Am. Chem. Soc.* **142**, 222–232 (2020).
20. Davydov, A., Davydov, R., Gräslund, A., Lipscomb, J. D. & Andersson, K. K. Radiolytic Reduction of Methane Monooxygenase Dinuclear Iron Cluster at 77 K. *Journal of Biological Chemistry* **272**, 7022–7026 (1997).
21. Fox, B. G. *et al.* Moessbauer, EPR, and ENDOR studies of the hydroxylase and reductase components of methane monooxygenase from Methylosinus trichosporium OB3b. *J. Am. Chem. Soc.* **115**, 3688–3701 (1993).
22. Bradley, J. M. *et al.* Reaction of O_2 with a diiron protein generates a mixed-valent Fe^{2+}/Fe^{3+} center and peroxide. *Proc. Natl. Acad. Sci. U.S.A.* **116**, 2058–2067 (2019).
23. Thompson, J. W. *et al.* Structural and Molecular Characterization of Iron-sensing Hemerythrin-like Domain within F-box and Leucine-rich Repeat Protein 5 (FBXL5). *Journal of Biological Chemistry* **287**, 7357–7365 (2012).
24. LeGall, J. *et al.* Isolation and characterization of rubrerythrin, a non-heme iron protein from Desulfovibrio vulgaris that contains rubredoxin centers and a hemerythrin-like binuclear iron cluster. *Biochemistry* **27**, 1636–1642 (1988).
25. Abramson, J. *et al.* Accurate structure prediction of biomolecular interactions with AlphaFold 3. *Nature* **630**, 493–500 (2024).
